# Long-term trends and comparison of the burden of lower respiratory tract infections in China and globally from 1990 to 2021: an analysis based on the Global Burden of Disease study 2021

**DOI:** 10.3389/fpubh.2024.1507672

**Published:** 2024-12-10

**Authors:** Zhiwei Wang, Shuqi Meng, Yan Fan, Jianfeng Liu, Lina Zhao, Yan Cui, Keliang Xie

**Affiliations:** ^1^Department of Critical Care Medicine, Tianjin Medical University General Hospital, Tianjin, China; ^2^Department of Anesthesiology, Tianjin Institute of Anesthesiology, Tianjin Medical University General Hospital, Tianjin, China; ^3^Department of Pathogen Biology, School of Basic Medical Science, Tianjin Medical University, Tianjin, China

**Keywords:** lower respiratory infections, COVID-19 impact, disease burden, epidemiology, prediction mortality rates, incidence rates, disability-adjusted life years (DALYs)

## Abstract

**Background:**

This study aimed to describe the temporal trends in the age and sex burdens of lower respiratory infections (LRIs) in China and globally from 1990 to 2021 and to analyze their epidemiological characteristics to formulate corresponding strategies to control LRIs.

**Methods:**

This study utilized open data from the Global Burden of Disease (GBD) database from 1990 to 2021 to assess the burden of disease based on the prevalence, incidence, mortality, years lost (YLLs), years lived with disability (YLDs), and disability-adjusted life-years (DALYs) of LRIs in China and globally. Moreover, a comprehensive comparative analysis of the epidemiological characteristics of LRIs in China and globally was conducted via the Joinpoint regression model, age-period-cohort model (APC model), and stratified analysis of the study method from multiple dimensions, such as age, sex, and period. Finally, we used an autoregressive integrated moving average (ARIMA) model to predict the disease burden in LRIs over the next 15 years.

**Results:**

From 1990 to 2021, China's age-standardized incidence, deaths, and disability-adjusted life year (DALY) rates per 100,000 people decreased from 5,481.13 (95% CI: 5,149.05, 5,836.35) to 2,853.81 (95% CI: 2,663.94, 3,067.55), from 60.65 (95% CI. 52.96, 66.66) to 14.03 (95% CI: 11.68, 17) and from 3,128.39 (95% CI: 2,724.11, 3,579.57) to 347.67 (95% CI: 301.28, 402.94). The global age-standardized incidence, deaths, and DALY rates per 100,000 people, on the other hand, decreased from 6,373.17 (95% CI: 5,993.51, 6,746.04) to 4,283.61 (95% CI: 4,057.03, 4,524.89) and from 61.81 (95% CI: 56.66, 66.74) to 28.67 (95% CI: 25.92, 31.07) and from 3,472.9 (95% CI: 3,090.71, 3,872.11) to 1,168.8 (95% CI: 1,016.96, 1,336.95). The decline in the aforementioned indicators is greater in the female population than in the male population, and the decrease in China is more pronounced than the global trend. In China, the age-standardized incidence and mortality rates of LRIs showed an annual average percentage change (AAPC) of −2.12 (95% CI: −2.20, −2.03) and −4.77 (95% CI: −5.14, −4.39), respectively. Globally, the age-standardized incidence and mortality rates for LRIs decreased by −1.28 (95% CI: −1.37, −1.18) and −2.47 (95% CI: −2.61, −2.32). By 2036, the incidence of lower respiratory infections (LRI) among men and women in China is projected to decrease by 36.55 and 46.87%, respectively, while the mortality rates are expected to decline to 12.67% for men and increase by 71.85% for women. In comparison, the global decline in LRI incidence is lower than that observed in China, yet the reduction in mortality rates is greater globally than in China.

**Conclusions:**

Age-standardized incidence, mortality and disability-adjusted life years (DALYs) decreased more in China than at the global level between 1990 and 2021. Compared with the previous period, the COVID-19 pandemic has led to a significant decrease in the disease burden of LRIs. As the population continues to age, the disease burden of LRIs in the old adult population will become a major new public health challenge.

## Introduction

Lower respiratory tract infections (LRIs) caused by various pathogens, such as bacteria, viruses, and fungi, are among the leading causes of death globally and are characterized by clinically diagnosed pneumonia and bronchitis (1). According to the most recent published study from the Global Burden of Disease (GBD), it is estimated that there will be ~344 million cases of LRIs globally in 2021, with ~2.18 million deaths (2). The burden of LRIs is influenced mainly by a variety of associated factors, such as age, sex, pathogen type, and socioeconomic and environmental factors (3–6).

With improvements in medical conditions and the implementation of targeted preventive measures, the number of LRI illnesses and deaths in China has shown a decreasing trend. However, the current burden of disease is still heavy, and the number of LRI deaths in China in 2019 will still reach 185,000. In addition, improvements in air quality and the reductions of smoking, drinking, and other bad habits in the population as risk factors for LRIs are also worthy of attention (7).

Despite ongoing research, a notable gap exists in understanding how recent global health crises, particularly the coronavirus disease 2019 (COVID-19) pandemic, have influenced LRI trends (8). The epidemiological impact of COVID-19 on lower respiratory infections (LRI) has been significant and multifaceted (9). The pandemic has led to a reassessment of LRI incidence, with some studies indicating that the surge in COVID-19 cases may have obscured the prevalence of other respiratory infections (2). Additionally, COVID-19 has affected mortality rates associated with LRIs, as the influx of COVID-19-related deaths may contribute to an overall increase in LRI mortality (10). The strain on healthcare systems due to the pandemic has resulted in delayed diagnoses and treatments for LRI patients, potentially exacerbating their conditions. Public health interventions implemented to combat COVID-19, such as social distancing and mask-wearing, have also had the unintended effect of reducing the transmission of other respiratory viruses, influencing LRI incidence rates (11). Furthermore, the long-term consequences of COVID-19, including potential lung damage, may increase the risk of future lower respiratory infections (12). Lastly, the rollout of COVID-19 vaccines may indirectly affect the epidemiology of other respiratory infections by enhancing population immunity (13). These dynamics underscore the need for a comprehensive understanding of the ongoing effects of COVID-19 on respiratory health (14).

Understanding the trends and burden of lower respiratory tract infections (LRIs) provides critical clues for epidemiology and public health control efforts. Epidemiological clues, such as patterns in incidence, mortality, and risk factors, are essential for identifying populations at higher risk, detecting emerging trends, and informing preventive measures. By analyzing the age-standardized incidence and mortality rates of LRIs, this study highlights the changes in disease burden over time, including the impact of the COVID-19 pandemic on LRI trends in China compared to global patterns. This study aims to fill this gap by examining long-term trends in the burden of LRIs in China from 1990 to 2021, focusing on the changes brought about by the COVID-19 pandemic. This study presents a description and analysis of the incidence, prevalence, mortality, years lost (YLLs), years lived with disability (YLDs), and disability-adjusted life years (DALYs) of LRIs in China and globally in 2021 based on just-released data from the Global Burden of Disease, Injury, and Risk Factors Study (GBD) 2021. We will investigate the etiology and risk factors of lower respiratory infections (LRIs) across different age groups, analyzing trends and future burdens using Joinpoint regression modeling, age-time-cohort analysis, stratified analyses, and autoregressive integrated moving average (ARIMA) modeling. This comprehensive approach aims to inform the development of targeted prevention strategies for LRIs.

## Methods

### Data sources overview

Data for this study were extracted from the Global Burden of Disease (GBD) 2021 website (https://vizhub.healthdata.org/gbd-results/), which includes data on 371 diseases and injuries and 88 risk factors from 204 countries and territories (7, 15). Specifically, we downloaded age-specific data on LRI, including prevalence, incidence, mortality, years of life lost (YLLs), years of life lived with disability (YLDs), and disability-adjusted life-years (DALYs), and age-standardized rates corresponding to the above metrics, from 1990 to 2021 in China and globally, with case definitions of LRI meeting the clinical criteria of the International Classification of Diseases 9 and 10 clinical criteria. To our knowledge, after raw data are obtained through national and international health surveys, hospital records, and death registries, the Institute for Health Metrics and Evaluation (IHME) at the University of Washington standardizes and cleanses the data to improve comparability and accuracy. Researchers also estimate epidemiologically relevant indicators of disease by etiology, sex, year, and location by implementing several different models, including the pooled model of deaths (CODEM), spatiotemporal Gaussian process regression (ST-GPR), and Bayesian meta-regression tool, Dimod-MR (1). In addition, the data extraction process may also need to take into account the impact of regional differences and temporal variations on the authenticity and reliability of the data.

### Join-point regression model

We utilized a Joinpoint regression model to evaluate trends in the disease burden of lower respiratory tract infections in China over different periods. The Joinpoint regression model is a statistical technique used to identify significant changes (or “joinpoints”) in trends over time. Joinpoints represent points where the direction or rate of change shifts, allowing for a more nuanced analysis of data trends. To determine these joinpoints, we use statistical criteria that assess the fit of the model at various potential points, selecting those that significantly improve the model's explanatory power. The choice of specific years as joinpoints is based on observed data trends and statistical testing, ensuring that the identified changes are both meaningful and representative of underlying shifts in the epidemiology of lower respiratory infections (16). The traditional regression model mainly fits and evaluates the overall trend of disease distribution within the study timeframe from a global perspective and cannot present the local change characteristics. This method, on the other hand, effectively avoids the non-objectivity of typical linear trend-based analysis and thus realizes the characterization and analysis of disease variation features specific to different time intervals on a global time scale. The annual percent change (APC), average annual percent change (AAPC), and 95% CI are the main outcome metrics of the Joinpoint model. APC is used to evaluate the internal trend of the independent intervals of the segmentation function or the global trend when the number of joinpoints is 0, whereas AAPC is used to comprehensively evaluate the global mean trend of change that encompasses multiple intervals. The parameters of AAPC are calculated by weighting the regression coefficients of the intervals by the width of the span of the segmented intervals w. Joinpoint (version 5.1.0; National Cancer Institute, Rockville, Maryland, USA) was used to create this model.

### Age-period-cohort model

The APC model further decomposes the effects of age, period, and cohort, allowing for analysis of age-group-specific incidence changes over time and the influence of generational shifts on LRI risk. The purpose of age-period-cohort analyses is to assess the impact of age, period, and cohort effects on outcomes (e.g., disease incidence or mortality). Age effects represent the differential risk of outcomes associated with different age groups; period effects represent the impact of temporal changes on outcomes across all age groups; and cohort effects represent variations in outcomes between groups of individuals with the same year of birth (cohorts) as a result of early life experiences and social factors. The general log-linear form of the age-period-cohort model is as follows:


$$\begin{array}{l} \log\left(M\right)=\text{μ}+{\text{α}}_{\text{i}}\;*age+{\text{β}}_{\text{j}}\;*period+{\text{γ}}_{\text{K}}\;*\;cohort+\text{ε} \end{array}$$

where μ represents the intercept error, ε represents the random error, and α, β, and γ represent the age effect, period effect, and cohort effect, respectively (17). In this study, we extracted the incidence data of lower respiratory tract infections in China and globally from the GBD2021 database from 1990 to 2021 and divided them into 20 age groups according to the groups of every 5 years (< 5, 5–9, 10–14.....80–84, 85–89, >90) into 20 age groups; 1990–2021 were divided into one period group according to every 5 years: 1992–1996, 1997–2001.....2017–2021 (because 1990–1991 was < 5 years, it could not be divided and could only be divided into 1992–2021), a total of 6-period groups were obtained; at the same time, the number of people in different age groups with lower respiratory tract infections in China from 1990 to 2021 was obtained by downloading from the GBD 2021 results tool. Since the annual population data provided by GBD were all single-year data, we averaged and combined them into 5-year intervals. This makes estimating a unique set of effects for each age, period, and cohort difficult because of the linear relationship between age, period, and cohort, and the problem of non-identifiability may still exist. The strong covariance between period, age, and cohort can be addressed by the intrinsic estimator (IE) algorithm (18), which was implemented in this study via the APC model analysis tool available on the National Cancer Institute (NCI) website (https://analysistools.cancer.gov/APC/). Net Drift (% per year) represents the overall time trend in mortality/morbidity, taking into account both components of the trend attributable to period and cohort factors. The APC model also estimated the time trend in mortality within each age group, expressed as the annual percentage change in age-specific mortality (Local Drift, % per year), which reflects trends in birth cohort effects and whose Wald chi-square test results suggest the presence or absence of birth cohort effects. Analysis of longitudinal age curves reveals whether there is an age effect; hypothesis testing of period deviation (period deviation) and the period rate ratio (period RR) reveals whether there is a period effect (19).

### Autoregressive integrated moving average model

The Autoregressive Integrated Moving Average (ARIMA) model is employed to forecast future trends in lower respiratory infections (LRIs). It combines the features of autoregressive (AR), sliding average (MA), and integral (I) models for describing and forecasting time series data. Autoregression (AR) is a model that assumes that the current values are a linear combination of several past values. It is denoted as AR(p), where p is the order of the autoregressive term. Integration (I) is used to make the time series smooth. This is achieved by performing a different operation on the non-smooth series. It is denoted as I(d), where d is the number of differencing operations used to smooth the series. The sliding average (MA) is the model's assumption that the current value is a linear combination of several past error terms. It is denoted as MA(q), where q is the order of the sliding average term. This statistical method is particularly effective for time series data, as it captures patterns and dependencies within historical data to predict future values (20). By integrating autoregressive terms (which relate current values to past values), differencing (to ensure stationarity), and moving averages (which smooth out fluctuations), the ARIMA model provides a robust framework for understanding underlying trends. In this study, we utilize ARIMA to estimate the future burden of LRIs, allowing for informed projections that can guide public health interventions and resource allocation.

### Statistical analysis

We analyzed the incidence, prevalence, mortality, DALYs, YLDs, and YLLs of lower respiratory infections (LRIs) in China and globally for 2021, along with their age-standardized rates. In this study, the reported mortality rates specifically refer to LRI-related deaths, distinguishing them from all-cause mortality. This focus allows for a more accurate assessment of the impact of lower respiratory infections on health outcomes. Using Joinpoint software (National Cancer Institute, Rockville, MD, USA), we calculated the average annual percentage change (AAPC) and corresponding 95% confidence intervals (95% CI) to illustrate the trends in LRI burden from 1990 to 2021. An AAPC with a 95% CI >0 indicates an increasing trend, a value < 0 indicates a decreasing trend and a value of 0 indicates a stable trend. For the age-period-cohort analysis, we divided the data into consecutive 5-year intervals from 1992 to 2021. Data from 1990 to 1991 were excluded because of an incomplete 5-year span. Age groups were categorized into 20 groups, ranging from under 5 years to over 95 years. To develop the ARIMA model, we first stabilized the time series data through differencing. The optimal model was selected based on the Akaike information criterion (AIC) via the Akaike() function. This approach ensures a comprehensive analysis of LRI trends over time, providing valuable insights into the shifting disease burden in China and globally. Statistical analysis and visualization were performed via R software (version 4.1.3) and Joinpoint software (version 4.9.1.0). A *p*-value of < 0.05 was considered statistically significant.

## Results

### Descriptive analysis of the Chinese and global LRIs in 1990 and 2021

In China, the number of LRI cases in 2021 was 44,704,579, and the number of deaths was 206,930, which were 12.35% and 56.43% lower than the 1990 data (50,936,862 cases and 474,883 cases), respectively. The global number of LRI cases in 2021 (343,606,787 cases) was 8.66% greater than that in 1990 (313,864,642 cases). However, the number of deaths (2,183,001 cases) decreased by 27.56% compared with that in 1990 (3,013,349 cases). This may be attributed to China's more stringent isolation strategy during the COVID-19 pandemic.

The age-standardized results of the prevalence, incidence, mortality, DALYs, YLDs and YLLs of LRIs in China in 2021 were as follows: 64.32 cases per 100,000 people, 2,853.81 new cases per 100,000 people, 14.03 deaths per 100,000 people, 347.67 disability-adjusted life years (DALYs)/per 100,000 people, 3.79 YLDs/per 100,000 people, 343.88 YLL/per 100,000 people. Compared with that in 1990, the decline in each of the above indicators in China exceeded the global decline over the same period, as shown in Tables 1, 2. We compared the above indicators with the disease burden indicators for China and the world in 2019, the year before the pandemic began (Supplementary Table 1), and found that the changes in these indicators in China after the onset of the pandemic were greater than those before the pandemic and also greater than the corresponding changes globally during the same period. This demonstrates that the impact of the COVID-19 pandemic on the disease burden of LRIs in China was greater than its impact on the global LRI burden.

**Table 1:** All-age cases and age-standardized prevalence, morbidity, mortality, YLL, YLD, and DALY rates of lower respiratory tract infections in China and global, 2021.

**Location**	**Measure**	**All-ages cases**			**Age-standardized rates per 100,000 people**		
		**Total**	**Male**	**Female**	**Total**	**Male**	**Female**
China	Prevalence	1,002,599 (941,423, 1,072,748)	522,210 (488,927, 558,476)	480,390 (449,471, 516,103)	64.32 (60.24,69.07)	70.11 (65.67,75.23)	60.17 (56.1,65.02)
	Incidence	44,704,579 (41,780,823, 47,783,965)	23,297,710 (21,689,677, 24,928,266)	21,406,869 (19,956,562,23,040,767)	2,853.81 (2,663.94, 3,067.55)	3,118.18 (2,914.12, 3,353.68)	2,664.57 (2,485.61, 2,870.23)
	Deaths	206,930 (171,261, 251,990)	119,076 (97,962, 143,881)	87,854 (64,860, 123,117)	14.03 (11.68,17)	20.45 (17.14,24.06)	10.31 (7.9,14.07)
	DALYs (Disability-Adjusted Life Years)	4,106,779 (3,504,141,4,808,453)	2,481,759 (2,056,424, 2,955,606)	1,625,020 (1,289,961, 2,153,679)	347.67 (301.28,402.94)	445.7 (379.02,515.46)	271 (223.85,331.31)
	YLDs (Years Lived with Disability)	58,602 (40,007,79,758)	30,785 (20,844, 42,328)	27,817 (18,976, 37,888)	3.79 (2.6,5.22)	4.13 (2.82,5.7)	3.54 (2.42,4.91)
	YLLs (Years of Life Lost)	4,048,177 (3,447,802,4,748,477)	2,450,974 (2,027,324, 2,921,449)	1,597,203 (1,259,685, 2,128,322)	343.88 (296.64,399.78)	441.57 (375.37,511.43)	267.46 (220.41,328.13)
Global	Prevalence	7,569,580 (7,198,813,8,005,307)	4,043,704 (3,839,119, 4,291,560)	3,525,875 (3,357,670, 3,727,671)	94.42 (89.78,99.84)	104.9 (99.6,111.09)	85.02 (81.05,90.13)
	Incidence	343,606,787 (325,214,314, 363,517,285)	183,254,768 (173853205, 194137698)	160,352,019 (151,438,078, 169,599,997)	4,283.61 (4,057.03, 4,524.89)	4,754.15 (4,510.74, 5,032.1)	3,861.73 (3,647.21, 4,080.66)
	Deaths	2,183,001 (1,979,915, 2,360,084)	1,172,230 (1,078,493, 1,269,920)	1,010,771 (878,596, 1,128,327)	28.67 (25.92,31.07)	34.25 (31.42,37.05)	24.5 (21.24,27.1)
	DALYs (Disability-Adjusted Life Years)	82,534,841 (72,611,990, 93,402,507)	45,402,004 (39,793,008, 51,982,463)	37,132,837 (31,972,682, 41,568,754)	1,168.8 (1,016.96, 1,336.95)	1,296.46 (1,128.84, 1,490.73)	1,054.29 (898.51, 1,194.39)
	YLDs (Years Lived with Disability)	448,180 (303,955, 616,812)	241,260 (164,111, 332,424)	206,921 (140,695, 283,870)	5.6 (3.8,7.73)	6.24 (4.25,8.58)	5.02 (3.41,6.94)
	YLLs (Years of Life Lost)	82,086,660 (72,180,867, 92,994,856)	45,160,744 (39,456,234, 51,754,976)	36,925,916 (31,807,749, 41,375,899)	1,163.2 (1,010.98, 1,330.41)	1,290.22 (1,121.14, 1,483.36)	1,049.26 (893.64, 1,188.58)

**Table 2:** All-age cases and age-standardized prevalence, morbidity, mortality, YLL, YLD, and DALY rates of lower respiratory tract infections in China and global, 1990.

**Location**	**Measure**	**All-ages cases**			**Age-standardized rates per 100,000 people**		
		**Total**	**Male**	**Female**	**Total**	**Male**	**Female**
China	Prevalence	1,141,642 (1,068,391, 1,221,122)	578,411 (541,577, 620,165)	563,231 (526,566, 605,248)	122.23 (115.1, 129.72)	124.96 (117.73, 132.62)	121.02 (113.75, 129)
	Incidence	50,936,862 (47,531,532, 54,596,483)	25,866,749 (24,096,969, 27,685,433)	25,070,113 (23,395,687, 26,890,639)	5,481.13 (5,149.05, 5,836.35)	5,621.89 (5,285.09, 5,997.34)	5,411.27 (5,073.27, 5,786.22)
	Deaths	474,883 (414,631, 532,255)	257,238 (225,826, 292,396)	217,646 (181,268, 246,830)	60.65 (52.96,66.66)	71.32 (64.39,78.39)	54.51 (43.4,61.45)
	DALYs (Disability-Adjusted Life Years)	32,576,310 (28,139,744, 37,498,333)	17,973,677 (15,333,113, 20,702,436)	14,602,633 (12,606,137, 16,856,157)	3,128.39 (2,724.11, 3,579.57)	3,340.53 (2,883.47, 3,810.16)	2,946.21 (2,542.5, 3,390.86)
	YLDs (Years Lived with Disability)	69,365 (46,567, 97,270)	35,241 (23,527, 49,469)	34,125 (22,781, 47,749)	7.29 (4.91, 10.09)	7.46 (4.97, 10.3)	7.22 (4.85, 10.06)
	YLLs (Years of Life Lost)	32,506,945 (28,076,659, 37,421,390)	17,938,436 (15,294,863, 20,669,931)	14,568,508 (12,575,991, 16,832,087)	3,121.09 (2,717.19, 3,569.78)	3,333.07 (2,875.38, 3,802.42)	2,938.99 (2,534.59, 3,385.7)
Global	Prevalence	6,952,663 (6,531,632, 7,404,351)	3,698,589 (3,473,023, 3,941,273)	3,254,074 (3,050,362, 3,462,417)	140.56 (132.94,148.87)	156.71 (148.49,165.73)	127.86 (120.43,135.32)
	Incidence	313,864,642 (294,055,265, 333,297,862)	166,991,424 (156,457,644, 177,508,398)	146,873.218 (137,447,466, 156,213,059)	6,373.17 (5,993.51, 6,746.04)	7,115.52 (6,694.46, 7,528.04)	5,790.87 (5,458.63, 6,145.5)
	Deaths	3,013,349 (2,744,313, 3,291,759)	1,573,352 (1,424,678, 1,731,386)	1439997 (1286170,1590813)	61.81 (56.66,66.74)	70.02 (64.82,75.26)	56.37 (50.55,61.63)
	DALYs (Disability-Adjusted Life Years)	204,174,057 (180,683,529, 229,006,748)	106,820,687 (93,919,782, 121,095,966)	97,353,370 (84,903,574, 110,194,838)	3,472.9 (3,090.71, 3,872.11)	3,623.79 (3,227.44, 4,055.43)	3,352.94 (2,942.6, 3,781.51)
	YLDs (Years Lived with Disability)	418,274 (283,296, 583,462)	223,195 (151,343, 311,266)	195,079 (132,226, 271,019)	8.36 (5.68, 11.6)	9.3 (6.33, 12.88)	7.6 (5.16, 10.53)
	YLLs (Years of Life Lost)	203,755,783 (180,293,489, 228,567,303)	106,597,492 (93,727,649, 120,801,448)	97,158,291 (84,725,662, 110,010,634)	3,464.54 (3,081.01, 3,862.65)	3,614.49 (3,219.03, 4,044.71)	3,345.34 (2,933.26, 3,773.84)

**Figure 1:**
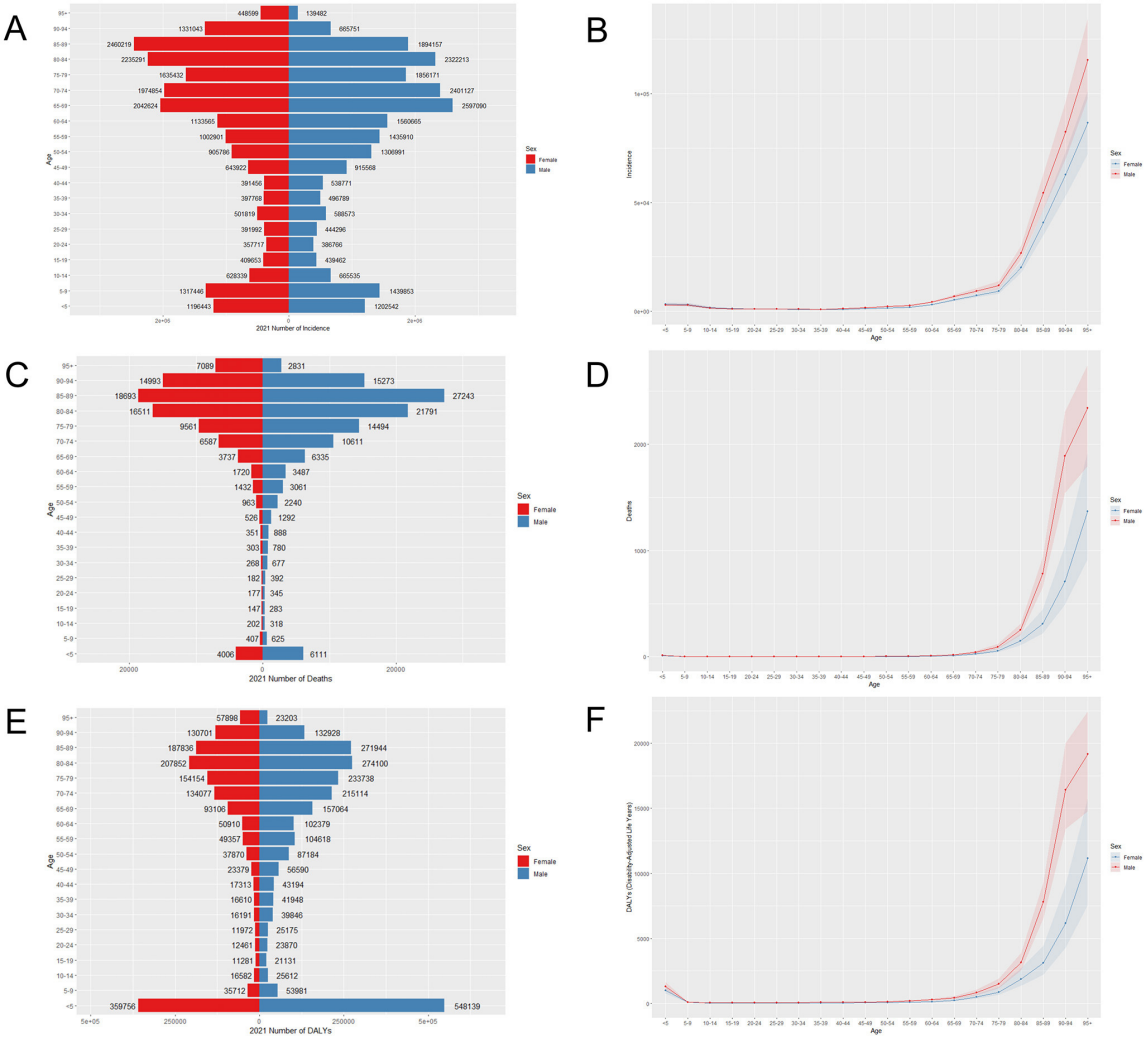
Age-specific incidence **(A)**, mortality **(C)**, DALY **(E)**, and age-standardized rates **(B, D, F)** of LRIs in China in 2021.

**Figure 2:**
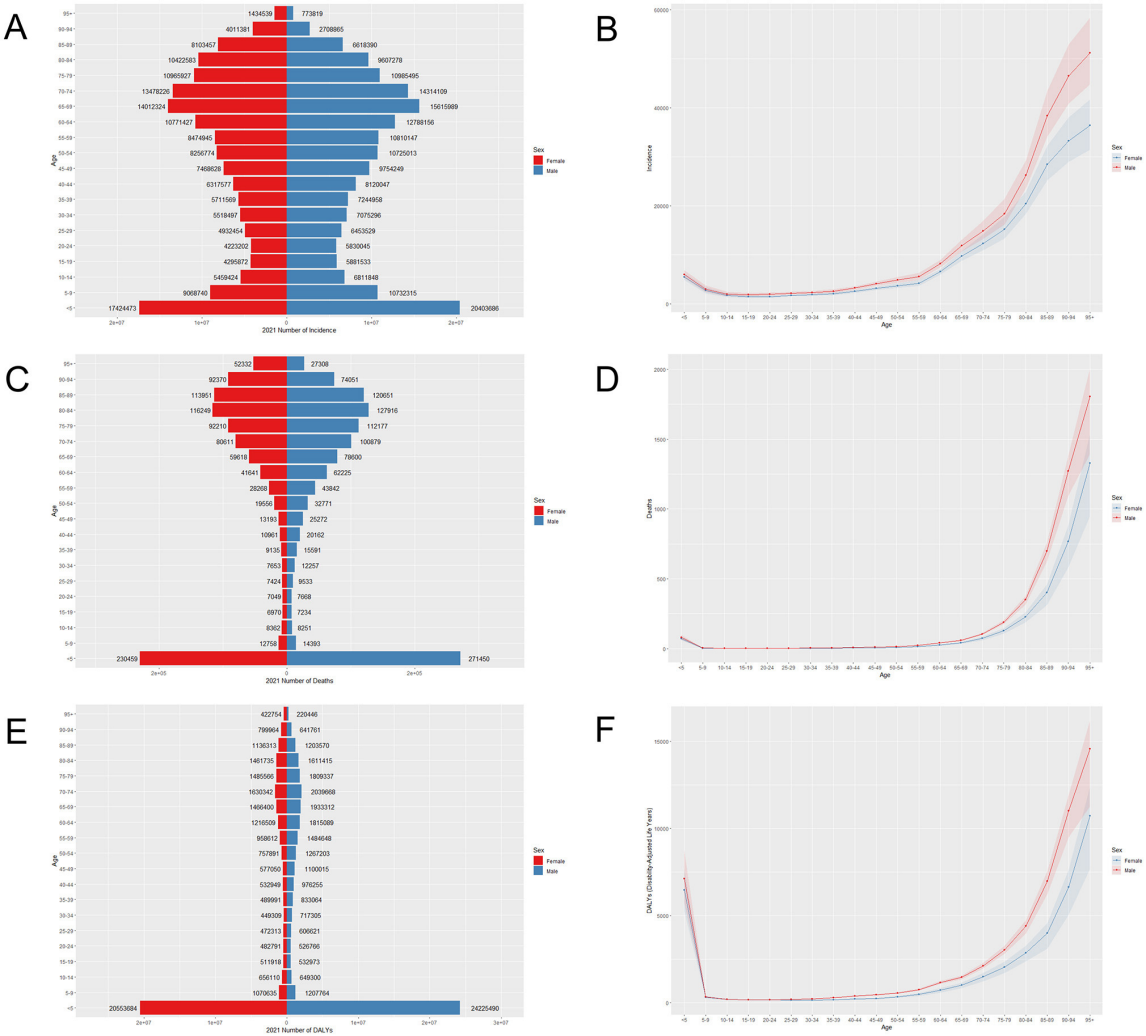
Age-specific incidence **(A)**, mortality **(C)**, DALY **(E)**, and age-standardized rates **(B, D, F)** of LRIs globally in 2021.

The population pyramid is used to visualize the prevalence, incidence, and mortality proportion in different age groups to the corresponding total number of people. Figure 1 shows the incidence, mortality, and DALYs of LRIs for different sexes and age groups in China in 2021. LRIs occur more frequently in people under 10 and over 60 years of age than in other age groups, with a rapid increase in the number of cases between 50 and 94 years of age. The highest incidence rates are among men aged 85–89 years and women aged 65–69 years. A similar trend is observed concerning deaths; the highest number of deaths for both males and females is concentrated between the ages of 85 and 89, with a sharp increase after the age of 65. The highest number of DALYs for males and females was concentrated in the < 5-year age group, with a gradual increase in the number of DALYs in the 60–85-year age range. The number of incidences and deaths in males is greater than that in females in most age segments, with the number of morbidities in males being lower than that in females only at the age of 85 years and above, and the number of deaths and DALYs being greater in females than in males only in the age group of 95 years and above. This may be related to the difference in the ratio of men to women and the average life expectancy of men and women in China (21, 22). However, the distribution of the above indicators by age group in China in 1990 was such that the maximum number of incidences, deaths, and DALYs were concentrated in the < 5 years of age group. The trend of the disease burden of LRIs being greater for males than for females remains unchanged in China from 1990 to 2021, but the main burden of disease has shifted from children to old adults (Supplementary Figure 1). Globally, the highest number of morbidities, deaths, and DALYs in all age groups in 1990 and 2021 were among those < 5 years of age (Figure 2). This suggests that the disease burden of LRIs in children < 5 years of age is lower in China than in the rest of the world and that the COVID-19 epidemic has increased the disease burden of LRIs in the global old adult population. This suggests that socio-economic factors play an important role in the burden of LRIs. Key factors include increased income, which enhances access to healthcare and improves living conditions (23); widespread education, which boosts health literacy and disease prevention awareness (24, 25); and improved access to healthcare, which can influence timely diagnosis and treatment (26). Additionally, urban-rural disparities, environmental exposures (e.g., air pollution), and occupational risks further impact LRI rates (2, 27). These factors highlight the need for targeted public health interventions to address socio-economic inequalities and effectively reduce the burden on local healthcare systems.

**Figure 3:**
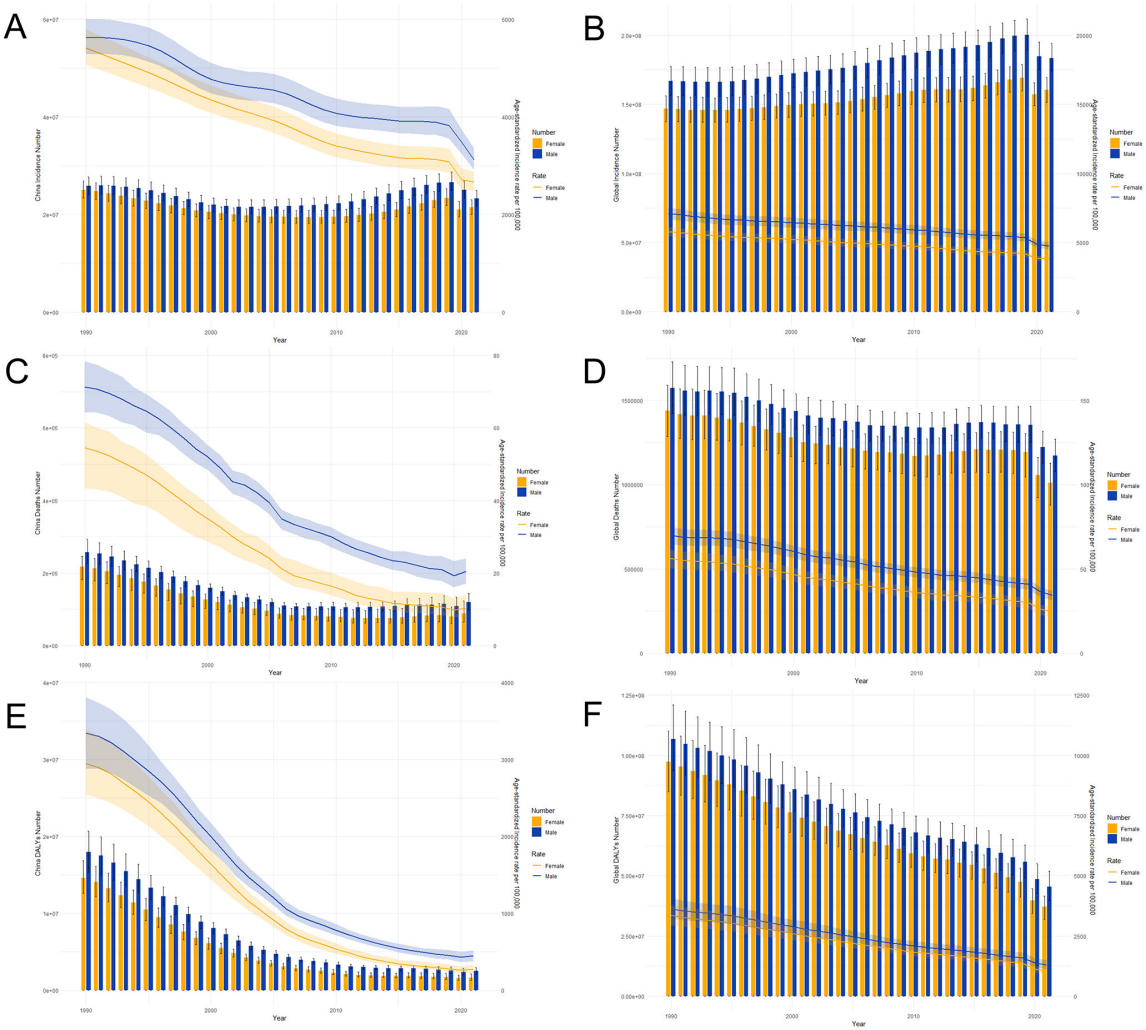
Trends in the number of LRIs by sex and age-standardized morbidity, mortality, and disability-adjusted life year (DALY) rates of LRIs in China **(A, C, E)** and globally **(B, D, F)** from 1990 to 2021.

Figure 3 shows the trends in the number of LRIs, deaths, DALYs by sex across all age groups, and age-standardized rates in China and globally for the period from 1990 to 2021. The incidence and number of deaths in China steadily increased after 2010, but the overall trends of incidence, mortality, and DALY age-standardized rates decreased annually. This may be because, compared with that in the previous year, the incidence rate in 2020 had a relatively large decrease, but the mortality rate increased compared with that in the previous year. Globally, the overall trends of the three indicators from 1990 to 2021 are roughly the same as those in China, but all of them clearly decrease in 2020. Although the disease burden of LRIs is decreasing annually, it remains to be seen whether the occurrence of an epidemic affects the current trend.

### Join-point analysis of LRI incidence and mortality in china and globally

**Figure 4:**
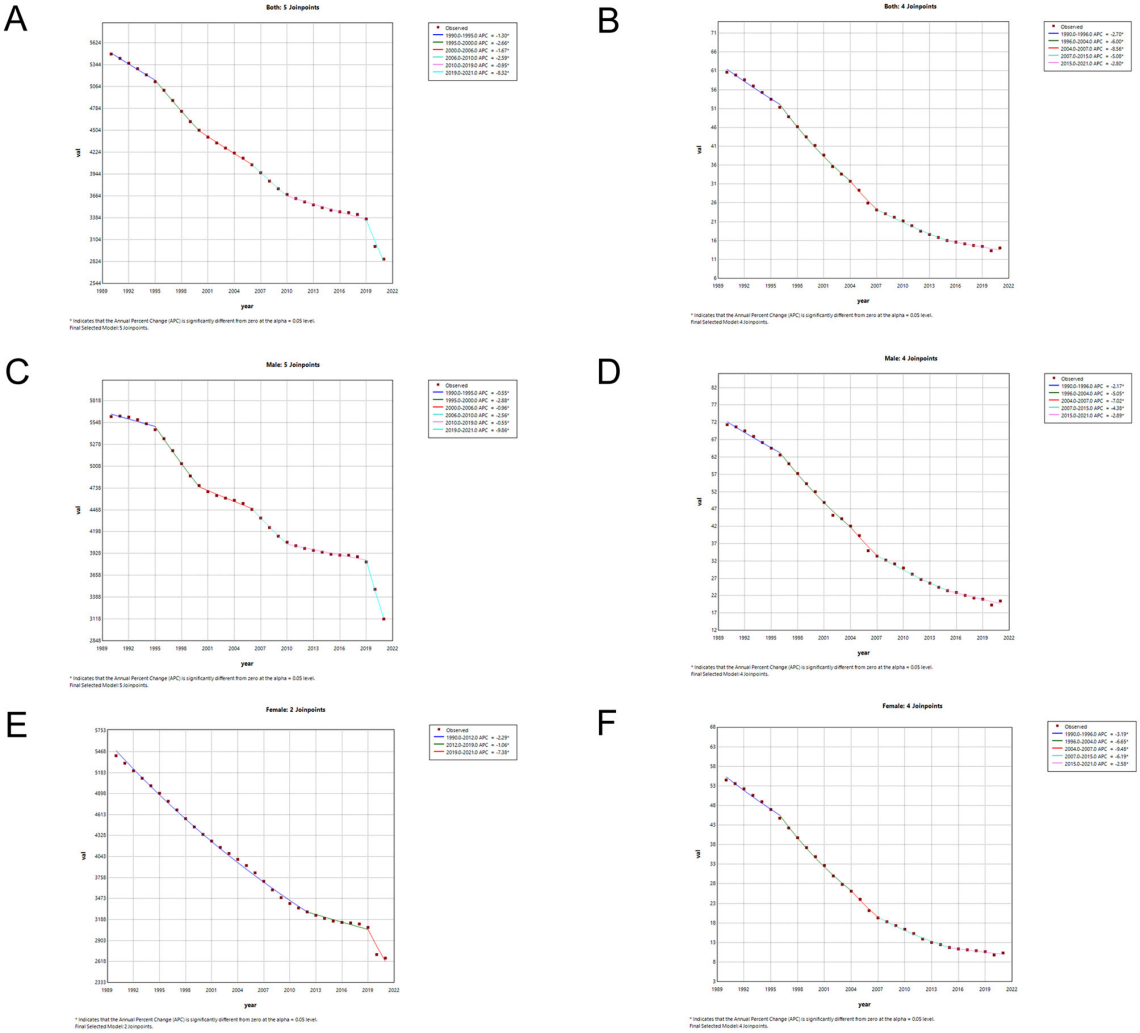
Joinpoint regression analysis of sex- and age-standardized incidence rates of LRIs in China **(A, C, E)** and globally **(B, D, F)** from 1990 to 2021.

**Figure 5:**
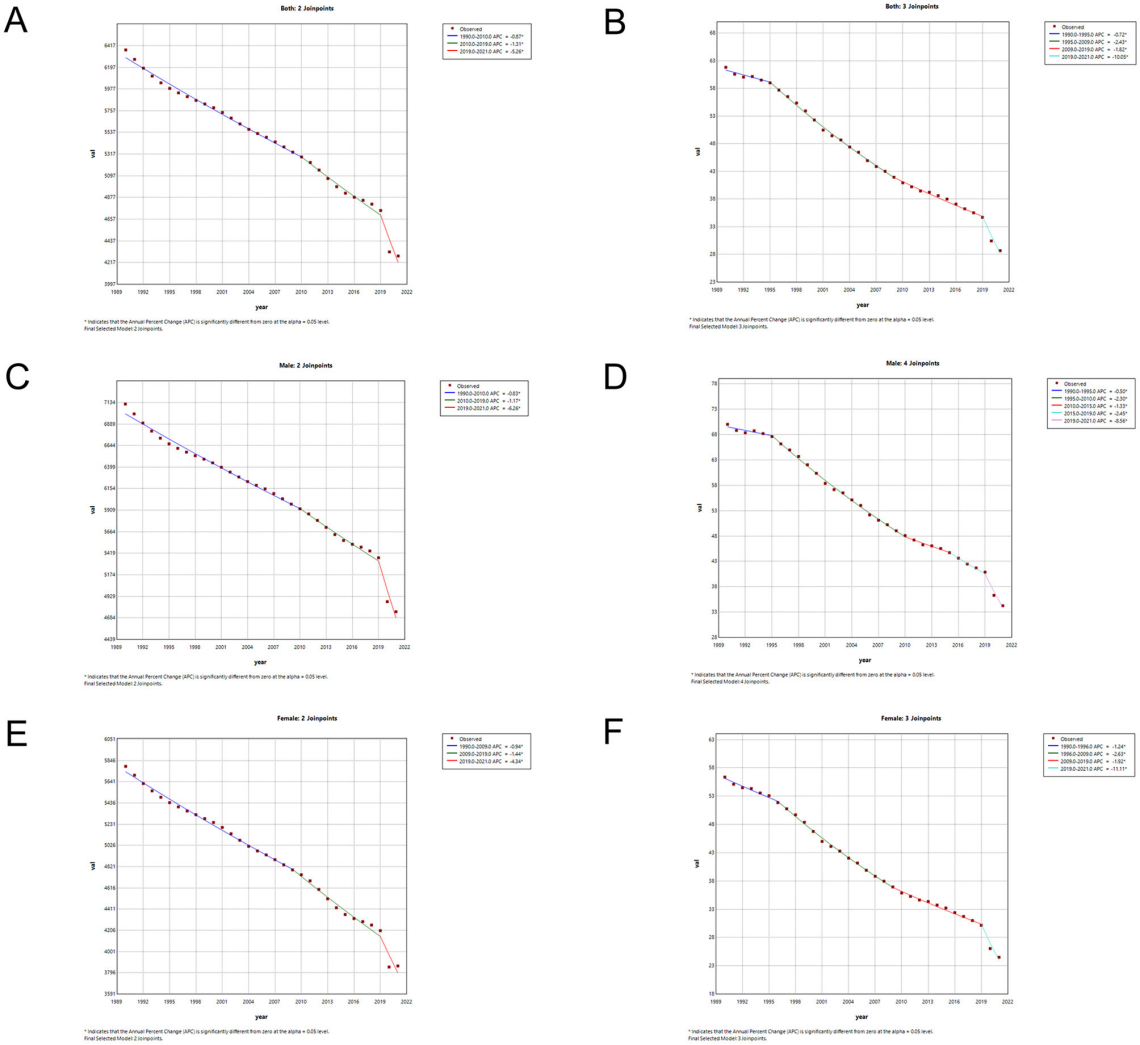
Joinpoint regression analysis of sex- and age-standardized mortality rates of LRIs in China **(A, C, E)** and globally **(B, D, F)** from 1990 to 2021.

The results of the Joinpoint regression analysis of the age-standardized incidence and mortality rates of LRIs in China from 1990 to 2021 are shown in Figures 4, 5. We found that, among males, the trend of disease incidence decreased significantly from 1995 to 2000 and from 2006 to 2010 for both periods [APC = −2.88 (1996–1999), 95% CI: −3.10, −2.65], [APC = −2.56 (2006–2010), 95% CI: −2.93, −2.19] for females, whereas the declining trend in incidence slowed from 2012 to 2019 [APC = −1.06 (1995–1999), 95% CI: −1.44, −0.68]. Globally, the declining trend of incidence rates for both sexes was relatively stable until 2019, after which the epidemic significantly reduced the incidence rates for both sexes in China and the world.

In terms of mortality rates of the LRIs, the mortality rates for both sexes in China maintained a relatively rapid declining trend. The age-standardized mortality rates exhibited the greatest declining trend from 2004 to 2007, with the highest rates for males (2004–2007 APC = −7.01, 95% CI: −9.84, −4.09) and females (2004–2007 APC = −9.485, 95% CI: −12.29, −6.58). The overall downward trend in the annual standardized rate of death globally until 2019 was lower than that in China, but there was a significant decrease in global mortality rates for both sexes after 2019, with a significant reduction for males (2019–2021 APC = −8.55, 95% CI: −10.41, −6.66) and females (2019–2021 APC = −11.11, 95% CI: −12.96, −9.22). Detailed information on the APC values can be found in Supplementary Table 2.

Table 3 shows the AAPC values for LRI incidence and mortality from 1990 to 2021. The age-standardized incidence and mortality rates of LRIs in China decreased by −2.12 (95% CI: −2.20, −2.03) and −4.77 (95% CI: −5.14, −4.39), respectively. The global age-standardized incidence and mortality rates of LRIs decreased by −1.28 (95% CI: −1.37, −1.18) and −2.47 (95% CI: −2.61, −2.32), respectively. The AAPC values for LRI incidence and mortality in China were lower than those in the world, and the AAPC values for both incidence and mortality were lower in women than in men.

**Table 3:** The AAPC values for LRI incidence and mortality in China and globally from 1990 to 2021.

		**Sex_name**	**Joinpoint model**	**AAPC index**	**Start Obs**	**End Obs**	**AAPC**	**AAPC C.I. low**	**AAPC C.I. high**	**Statistically significant (0 = No, 1 = Yes)**	**Test statistic**	***P*-value**
China	Incidence	Both	5	Full Range	1990	2021	−2.1223	−2.2048	−2.0396	1	−49.8291	< 0.001
		Female	2	Full Range	1990	2021	−2.3528	−2.5199	−2.1854	1	−27.2469	< 0.001
		Male	5	Full Range	1990	2021	−1.8928	−1.9781	−1.8075	1	−43.0788	< 0.001
	Death	Both	4	Full Range	1990	2021	−4.7704	−5.1439	−4.3954	1	−24.3769	< 0.001
		Female	4	Full Range	1990	2021	−5.3752	−5.7464	−5.0025	1	−27.5483	< 0.001
		Male	4	Full Range	1990	2021	−4.1018	−4.4709	−3.7314	1	−21.2896	< 0.001
Global	Incidence	Both	2	Full Range	1990	2021	−1.2837	−1.3785	−1.1888	1	−26.3628	< 0.001
		Female	2	Full Range	1990	2021	−1.3242	−1.4239	−1.2244	1	−25.8469	< 0.001
		Male	2	Full Range	1990	2021	−1.2886	−1.3922	−1.1849	1	−24.2127	< 0.001
	Death	Both	3	Full Range	1990	2021	−2.4723	−2.6197	−2.3247	1	−32.4414	< 0.001
		Female	3	Full Range	1990	2021	−2.7047	−2.8521	−2.5571	1	−35.45	< 0.001
		Male	4	Full Range	1990	2021	−2.2922	−2.4858	−2.0983	1	−22.921	< 0.001

### The effects of age, period, and cohort on incidence and mortality rates

**Figure 6:**
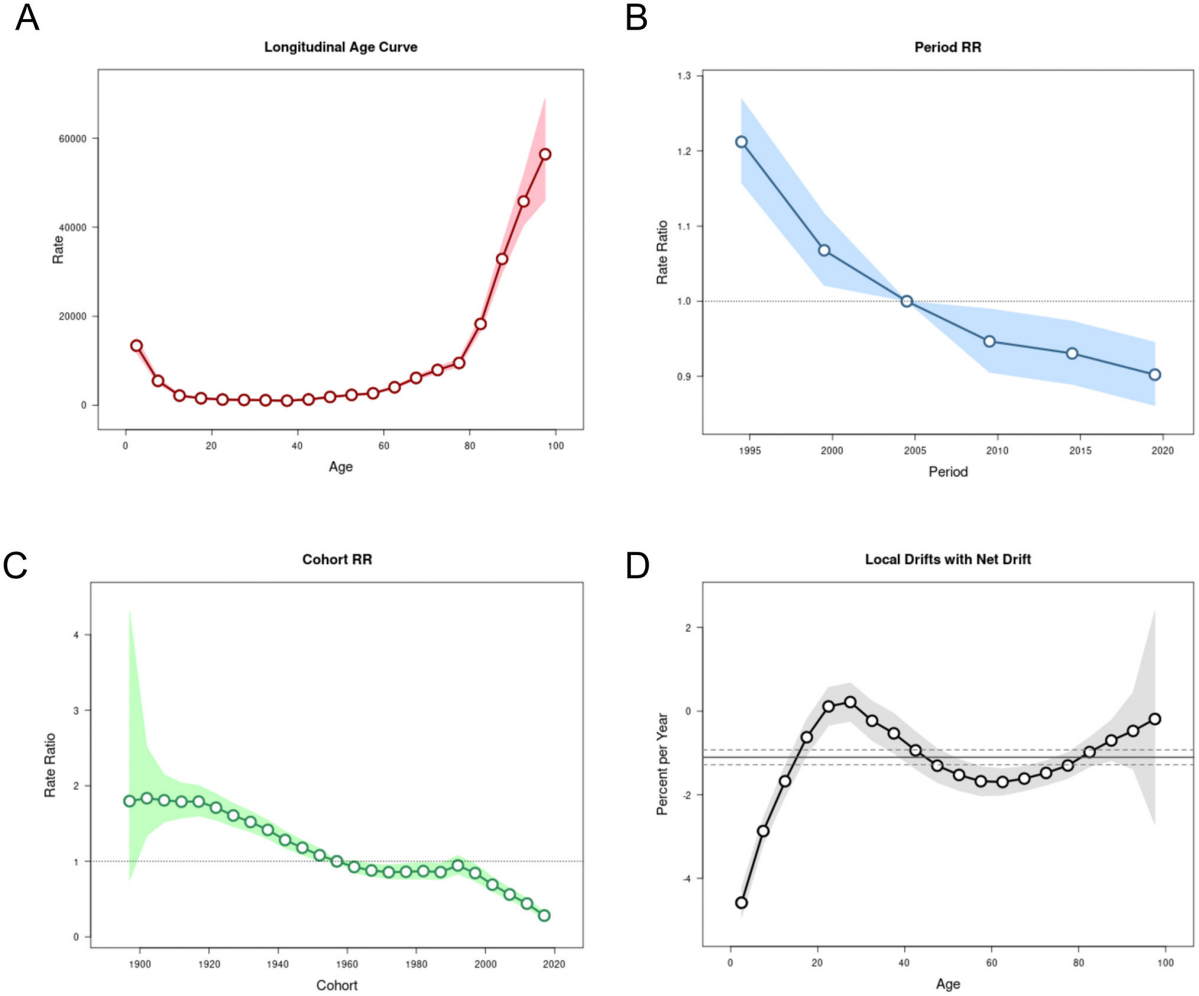
The APC model of LRI incidence in China. **(A)** Age effect. **(B)** Period effect. **(C)** Birth cohort effect. **(D)** Annual percentage change in age-specific mortality rates.

**Figure 7:**
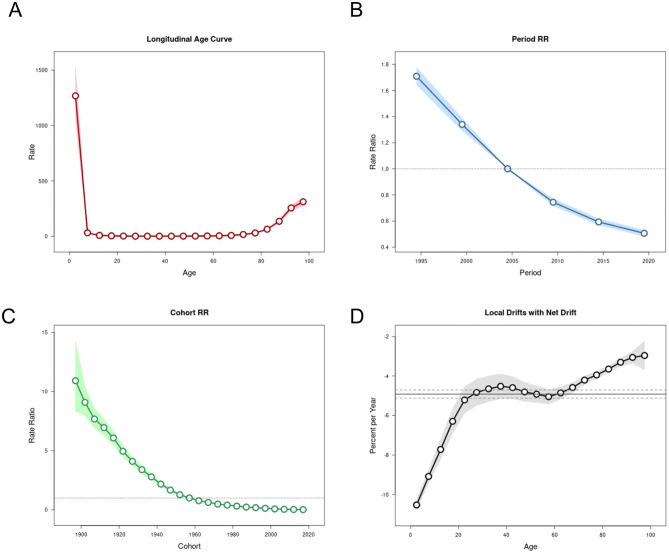
The APC model of LRI deaths in China. **(A)** Age effect. **(B)** Period effect. **(C)** Birth cohort effect. **(D)** Annual percentage change in age-specific mortality rates.

Figures 6, 7 depict the trends of LRIs and mortality rates in the APC model for the period of 1992–2021 in China. The net drift (% per year) values reflecting the overall trends of the two indicators are −1.1043 and −4.9181, which show that both are decreasing and that the mortality rate is decreasing more than the incidence rate. In terms of the effect of age, the incidence rate decreases gradually before the age of 12.5 years. It increases rapidly after the age of 62.5 years, whereas the mortality rate decreases gradually with age before the age of 12.5 years and increases rapidly with age after the age of 67.5 years. In terms of the period effect, after May 2004 to the present, the decline in incidence and mortality rates in China was significantly lower than before. Since the outbreak of SARS in 2003, comprehensive infectious disease control measures in China, including improvements in resources, systems, and legislation, have contributed to this downward trend. Key developments include the introduction of the **Regulations on Emergency Response to Public Health Emergencies** in 2003, the **Infectious Disease Prevention and Control Law of the People's Republic of China** in 2004, and subsequent updates in 2013 and 2020 (28). Regarding the cohort effect, the incidence and mortality rates of LRIs in the population born after 1957 were lower than those born before 1957. Historically, the Asian flu outbreak in China in 1957 prompted the government to strengthen public health infrastructure, including the establishment of the National Influenza Center and the publication of the Influenza Manual in 1958. These measures may have contributed to the observed differences in LRI incidence and mortality across population cohorts (29, 30).

### Decomposition analysis of drivers of LRI epidemiology

**Figure 8:**
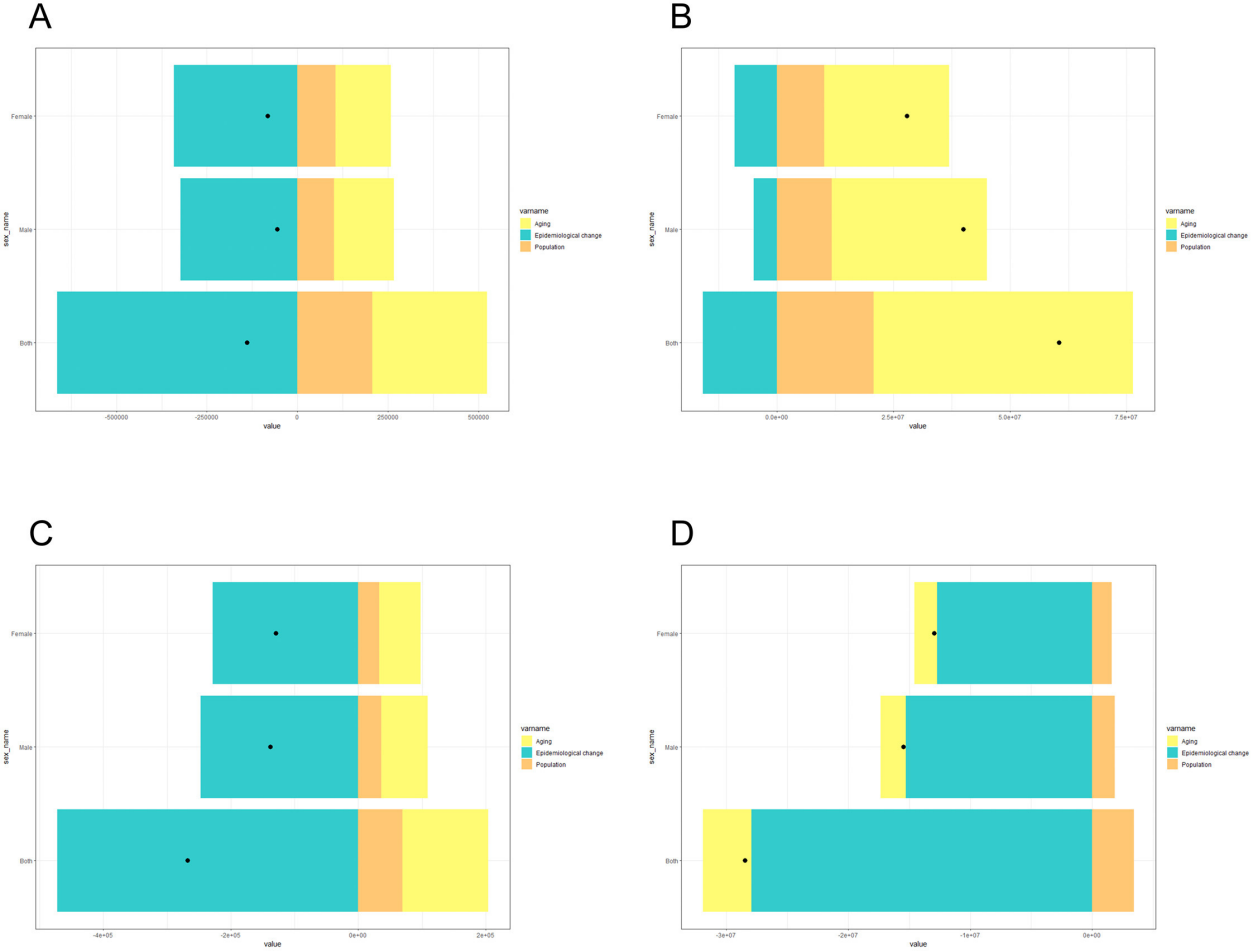
Decomposition analysis of the drivers of LRI incidence **(A)**, morbidity **(B)**, mortality **(C)**, and DALY **(D)** rates in China in 2021.

**Figure 9:**
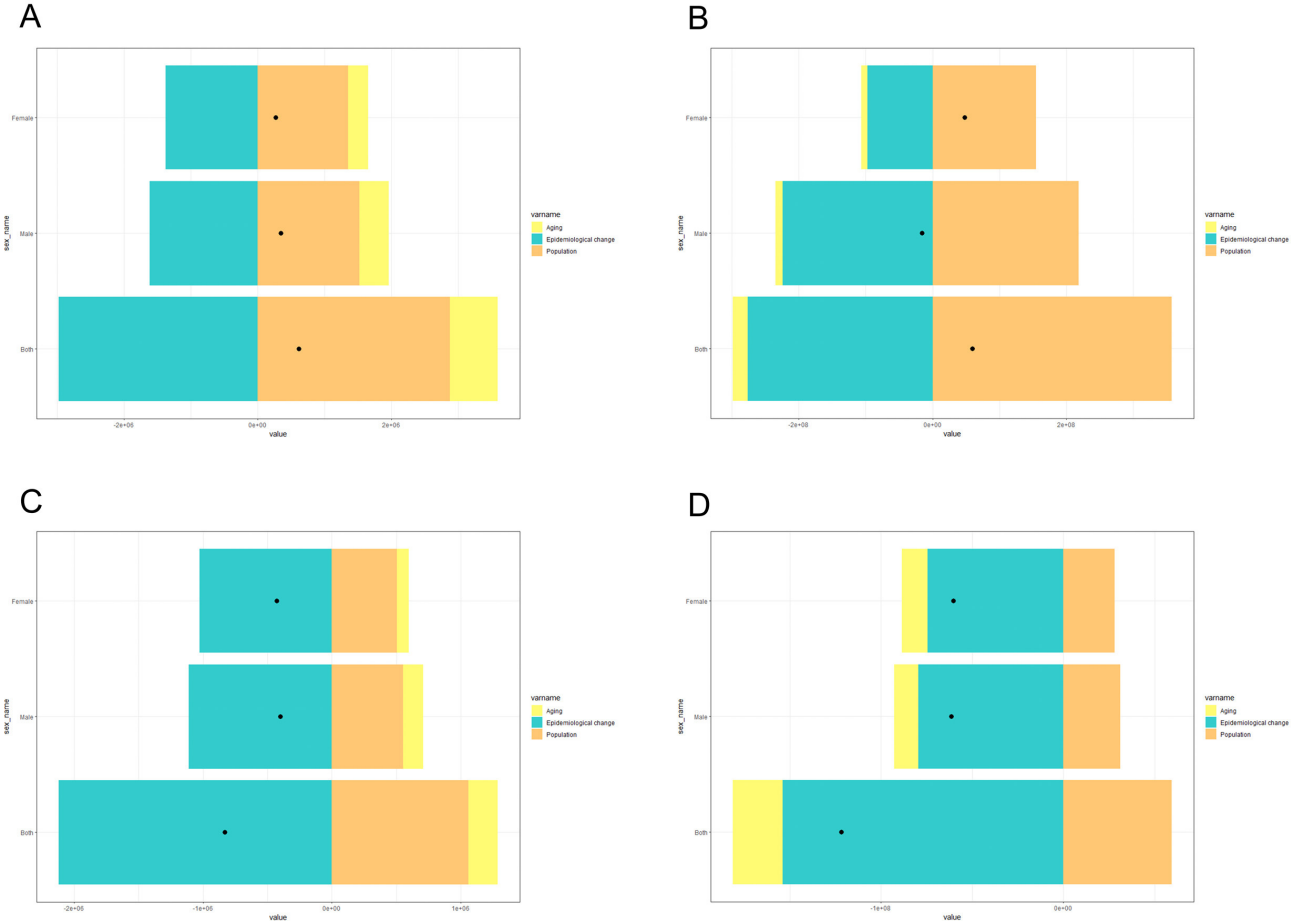
Decomposition analysis of the drivers of LRI incidence **(A)**, morbidity **(B)**, mortality **(C)**, and DALY **(D)** rates globally in 2021.

To assess the extent to which population aging, population size, and epidemiological changes affect the age-standardized prevalence, incidence, mortality, and DALY of LRIs in China and globally, we decomposed and analyzed the above data (Figures 8, 9); within China, the decrease in LRI incidence was most affected by population aging (92.31%), whereas age-standardized rates of prevalence, mortality, and DALY reduction were all most affected by epidemiologic changes (477.25, 282.56, and 98.11%, respectively). Worldwide, the increase in prevalence was most affected by epidemiological changes (−482.98%), the increase in incidence was most affected by population size (−1,113.53%), and the decrease in mortality and the age-standardized rate of DALYs was most affected by epidemiological changes (346.33, 126.37%; Table 4).

**Table 4:** Decomposition analysis of aging, population, and epidemiological change of LRI epidemiology.

		**Sex_name**	**Overll_difference**	**Aging effect**	**Population effect**	**Epidemiological change**	**Aging percent**	**Population percent**	**Epidemiological change percent**	**Val_1990**	**Val_2021**
China	Prevalence	Both	−139,042.76	317,086.989	207,446.89	−663,576.639	−228.05	−149.2	477.25	1,141,642.2	1,002,599.5
		Male	−56,201.8	164,900.447	101,613.23	−322,715.48	−293.41	−180.8	574.21	578,411.5	522,209.7
		Female	−82,840.96	152,483.2	105,891.651	−341,215.807	−184.07	−127.83	411.89	563,230.7	480,389.8
	Incidence	Both	57,401,829.65	52,989,010.71	20,416,539.98	−16,003,721.04	92.31	35.57	−27.88	50,936,862	44,704,579
		Male	38,795,413.38	32,223,849.69	11,658,477.97	−5,086,914.273	83.06	30.05	−13.11	25,866,749	23,297,710
		Female	25,998,144.93	25,147,956.14	9,959,962.664	−9,109,773.874	96.73	38.31	−35.04	25,070,113	21,406,869
	Death	Both	−227,732.58	310,926.297	104,811.73	−643,470.611	−136.53	−46.02	282.56	474,883.4	206,930.22
		Male	−56,132.79	174,457.777	53,893.734	−284,484.301	−310.79	−96.01	506.81	257,237.8	119,076.39
		Female	−125,143.04	155,972.313	54,304.181	−335,419.53	−124.64	−43.39	268.03	217,645.6	87,853.84
	DALY	Both	−28,469,530.71	−3,983,772.922	3,445,857.537	−27,931,615.33	13.99	−12.1	98.11	32,576,310	4,106,779
		Male	−15,491,917.44	−2,070,107.573	1,837,874.211	−15,259,684.08	13.36	−11.86	98.5	17,973,677	2,481,759
		Female	−12,977,613.28	−1,866,675.684	1,596,579.971	−12,707,517.56	14.38	−12.3	97.92	14,602,633	1,625,020
Global	Prevalence	Both	616,916.2	720,653.712	2,875,874.261	−2,979,611.778	116.82	466.17	−482.98	6,952,663	7,569,580
		Male	345,114.95	439,714.327	1,523,371.7	−1,617,971.076	127.41	441.41	−468.82	3,698,589	4,043,704
		Female	271,801.24	301,493.017	1,352,237.133	−1,381,928.906	110.92	497.51	−508.43	3,254,074	3,525,875
	Incidence	Both	−18,347,044.35	−44,296,247.48	204,299,575.8	−178,350,372.7	241.44	−1,113.53	972.09	313,864,642	343,606,787
		Male	−18,325,548.15	−22,448,608.9	114,650,272.2	−110,527,211.5	122.5	−625.63	603.13	166,991,424	183,254,768
		Female	−3,627,296.44	−20,533,028.65	92,007,353.28	−75,101,621.07	566.07	−2,536.53	2,070.46	146,873,218	160,352,019
	Death	Both	−669,448.84	387,594.917	1,261,428.658	−2,318,472.417	−57.9	−188.43	346.33	3,013,349	2,183,001
		Male	−264,662.21	267,818.09	653,681.84	−1,186,162.142	−101.19	−246.99	448.18	1,573,352	1,172,230
		Female	−370,206.24	158,195.888	623,593.156	−1,151,995.282	−42.73	−168.44	311.18	1,439,997	1,010,771
	DALY	Both	−121,639,216.3	−27,471,135.78	59,542,337.59	−153,710,418.1	22.58	−48.95	126.37	204,174,057	82,534,841
		Male	−61,418,683.29	−13,286,999.83	31,270,972.92	−79,402,656.37	21.63	−50.91	129.28	106,820,687	45,402,004
		Female	−60,220,532.96	−13,955,843.19	28,254,075.36	−74,518,765.13	23.17	−46.92	123.74	97,353,370	37,132,837

### Prediction of LRIs prevalence and mortality in the next decade

**Figure 10:**
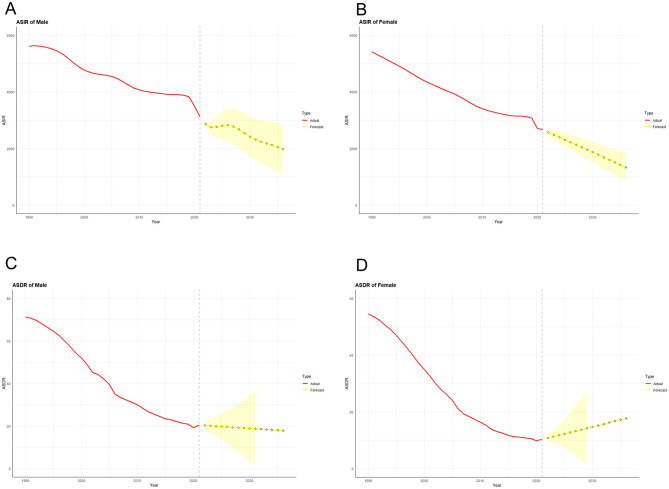
Predicted trends of LRI incidence **(A)** and mortality **(C)** in men and LRI incidence **(B)** and mortality **(D)** in women in China over the next 15 years (2022–2036). The red line represents the true trend of LRIs and mortality between 1990 and 2021; the yellow dotted line and shaded area represent the predicted trend and its 95% CI, respectively.

**Figure 11:**
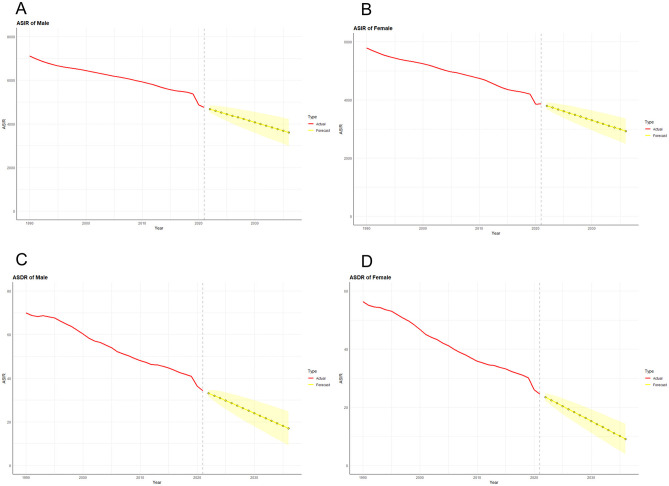
Predicted trends of LRI incidence **(A)** and mortality **(C)** in men and LRI incidence **(B)** and mortality **(D)** in women in Global over the next 15 years (2022–2036). The red line represents the true trend of LRIs and mortality between 1990 and 2021; the yellow dotted line and shaded area represent the predicted trend and its 95% CI, respectively.

On the basis of the available epidemiological data, an ARIMA model was used to predict the trend of morbidity and mortality associated with LRIs in both sexes in China and globally in the next 15 years (Figures 10, 11). After filtering by the auto.arima() function, the optimized models for the age-standardized incidence rates of LRIs in Chinese males and females were (0,1,0) and (2,1,0), respectively, with AIC values of 336.21 and 346.51. According to the prediction results, the age-standardized incidence rates of LRIs in Chinese males and females in 2,036 decreased by 36.55% compared with those in 2021 and by 49.87% to 1,978.483/100,000 and 1,335.520/100,000 people, respectively. The optimization models for the LRIs of the age-standardized mortality rates for males and females in China were (0, 2, 1) and (0, 2, 0), respectively, with AIC values of 84.66 and 41.38. The mortality of LRIs for males in China is projected to decrease by 12.67% to 17.854/100,000 people by 2,036, whereas the mortality for females will increase by 71.85% to 17.711/100,000 people.

Through the process described above, we also made predictions for future epidemiologic indicators related to LRIs globally. The ARIMA optimization models for the global age-standardized incidence rates for males and females were (0, 1, 0) and (0, 1, 1), respectively, with AIC values of 363.74 and 342.28. The global age-standardized incidence rates for males and females declined by 24.01% and 24.17% in 2036 to 3,611.556 and 2,928.278 per 100,000 population, respectively. The ARIMA optimization models for the world LRI age-standardized mortality rates for males and females were (0,1,1) and (0,1,0), with AIC values of 77.47 and 67.44, respectively. Compared with 2021, the global age-standardized mortality rates for males and females in 2036 decreased by 50.34 and 62.95%, respectively, to 17.005 per 100,000 and 9.076 per 100,000.

## Discussion

Based on the newly released GBD 2021 database, we comprehensively assessed the changes in LRI epidemiologic indicators (incidence, prevalence, mortality, DALYs, YLLs, YLDs) in China and globally from 1990 to 2021 and predicted the future trends of these indicators. We analyzed the epidemiological trends of LRIs in China and globally via Joinpoint analysis combined with APC modeling and decomposition analysis. Although Kang et al. published a study on the trends of mortality, etiology, and risk factors for LRIs in China based on data from the GBD2019 (31), with the pandemic COVID-19 outbreak, the corresponding measures taken worldwide for the prevention and treatment of LRIs have had a profound impact on the epidemiological changes in such diseases (8, 32, 33). Therefore, it is necessary to reevaluate and analyze the trends of epidemiological indicators of LRIs in China and the world after 2019 and to make appropriate predictions for possible future changes. This will facilitate the reformulation of targeted measures and the updating of effective interventions.

The results of the analysis revealed that although the incidence, prevalence, and YLD rates of the LRIs were higher in 2021 than in 1990 globally, the ASIR, ASPR, ASMR, ASDR, and ASYR (YLD and YLL) rates of the LRIs exhibited stable decreasing trends from 1990 to 2021 in both China and the world. In addition, the declines in the above indicators are more significant within China than globally, reflecting the fact that China's more stringent isolation and prevention strategies after SARS and COVID-19, as well as improvements in environmental pollution, especially air quality, have been effective in reducing the spread of respiratory diseases (34–36). Previous studies have similarly reported a decline in LRI incidence and mortality rates over recent decades, attributing these trends to improved healthcare access, vaccination coverage, and reductions in risk factors like smoking and air pollution (2, 27). In terms of age and sex, the burden of disease is greater in males than in females in most age groups, which correlates with sex-specific susceptibility to respiratory pathogens and with alcohol and tobacco use in males (5, 37–39). Notably, the main burden of disease for both the global and Chinese LRIs in 1990 was concentrated in children < 5 years of age. This status quo shifted in China in 2021 to a major burden concentrated in older adults >60 years of age, whereas globally, although the number of LRI morbidities and deaths in older adults has risen as a proportion of the all-age population, children < 5 years of age continue to occupy the first place for both indicators. This is consistent with previous studies showing a higher disease burden in this age group. This may be due to their immature immune system combined with exposure to new pathogens, as well as the increased probability of disease transmission in highly crowded school environments (40). The number of people over 65 in China in 2020 was 2.4 times greater than in 1990 (41). Moreover, the life expectancy of the Chinese population in 2020 will have increased by 8.7 years compared with that in 1990. This may reflect the aging of the Chinese population structure and the increasing proportion of government investment in child health management (42–44). In addition, older adults tend to exhibit an increased frequency of disease and poor prognosis due to functional decline of vital organs and a combination of comorbidities. Therefore, as populations continue to age around the world, especially in high-income countries, there is a need to continue to emphasize effective control of the burden of disease in children while focusing on preventing the causes and risk factors for LRIs in older adults and developing timely measures to reduce the fatal burden of LRIs (45, 46).

Joinpoint analysis revealed that the incidence of LRIs among men in China declined more sharply in 1995–2000 and 2006–2010 than in other periods did, whereas the decline in women's incidence was more moderate, which may be related to China's policy of accession to the Framework Convention on Tobacco Control (FCTC) and implementation of a nationwide anti-tobacco ban (47, 48). The APC model analysis revealed that the LRI incidence and mortality rates in China declined from 1990 to 2021. The effect of age on LRI morbidity and mortality is reflected in the fact that the trend of risk associated with children and old adults is much greater than that associated with other age groups. The incomplete development of the immune system in children and the decline in immune function in old adults promote susceptibility to LRIs, in addition to the associated decline in the organ system and the development of comorbidities in old adults (49–51). The achievements of the last 30 years in reducing the burden of disease in children with LRIs have provided us with valuable experience; children have higher vaccination rates against respiratory pathogens than do old adults do (52–54), but due to the decline in immune function, further evidence is needed to demonstrate whether vaccination has a protective effect equivalent to that of children (55). Moreover, vaccination is an important means of providing effective protection to the population, and more targeted vaccines need to be developed on the basis of the immunologic and epidemiologic characteristics of the older population (56–58). In addition, measures that are effective in pediatric populations, such as improving immune function by enhancing the nutritional status of the target population, could also be replicated in the old adult population (59). Overall, the burden of disease in childhood LRIs is currently concentrated in low-income countries, and with demographic changes in middle- and developed countries, more attention should be given to reducing the burden of disease in older populations (60). The increase in LRI burden, especially in the aftermath of the pandemic, signals a potential rise in respiratory infections due to the complex interplay of factors such as the resurgence of other respiratory pathogens, post-viral complications, and healthcare disruptions (61, 62). Policymakers should prioritize strengthening public health infrastructure to address these evolving challenges. This includes investing in robust surveillance systems to monitor trends in respiratory infections, improving access to healthcare services, and ensuring timely vaccination programs to reduce the burden of preventable diseases like pneumonia (63, 64).

The impact of the COVID-19 pandemic on global LRI morbidity and mortality has been significant. Owing to rigorous physical interventions such as universal mask use and comprehensive isolation policies, the spread of pathogens common to respiratory infections (e.g., influenza viruses and respiratory syncytial viruses) has been greatly limited (2, 65). Notably, despite the significant decline in LRI incidence and mortality globally after 2019, LRI mortality in China has increased slightly since the previous period and is mostly concentrated in the old adult population. As the first reported country of COVID-19, China's healthcare system was under tremendous pressure in the early stages of the outbreak, and the strain and skewing of healthcare resources may have been among the factors contributing to the increase in LRI mortality (66). The COVID-19 pandemic has had a profound impact on the epidemiology of lower respiratory tract infections (LRIs), reshaping trends in incidence, mortality, and healthcare access. Firstly, COVID-19 has led to changes in healthcare-seeking behaviors and healthcare accessibility. Many individuals delayed or avoided medical care during the pandemic due to lockdowns, healthcare facility restrictions, or fear of exposure, which likely reduced reported LRI cases and impacted timely diagnosis and treatment (67). Secondly, public health interventions, such as widespread mask-wearing, social distancing, and improved hygiene practices, significantly reduced the transmission of other respiratory pathogens, leading to a decrease in LRI incidence in some regions. For example, studies in urban centers in Asia and Europe documented a substantial drop in cases of influenza and other respiratory infections, which are often co-factors in LRI morbidity and mortality (68, 69). This temporary reduction, however, may not reflect longer-term trends, as these non-pharmaceutical interventions are lifted or adapted (70). Thirdly, COVID-19 has introduced new challenges in managing LRIs due to its potential long-term effects on lung health. Some COVID-19 survivors, especially those who experienced severe disease, may suffer from persistent respiratory issues such as lung fibrosis or reduced lung function, making them more susceptible to future LRIs (71, 72). This “long COVID” phenomenon represents a new cohort of individuals who could experience higher rates of LRIs, thus altering the epidemiological profile of LRIs post-pandemic (73). Together, these factors underscore the need for ongoing surveillance of LRI trends in the post-pandemic era. Further research is essential to understand how the pandemic's direct and indirect effects will continue to shape LRI epidemiology and to identify new strategies for managing this evolving burden.

Based on the above research findings, there is an urgent need for ongoing, robust monitoring of LRI trends, especially considering emerging respiratory pathogens and the long-term effects of COVID-19. Public health systems should strengthen data collection on LRI incidence, mortality, and risk factors, with a focus on vulnerable populations such as children, the old adults, and those with pre-existing health conditions. In particular, expanding surveillance at the regional level will provide more detailed data to inform localized interventions and ensure that high-risk populations receive timely care. Given the observed long-term respiratory effects in COVID-19 survivors, continued research into the “long COVID” phenomenon and its impact on LRI susceptibility is crucial. Policies should be developed to address post-viral care for severe COVID-19 recoverers, particularly with regard to lung health and the increased risk of secondary infections. This will help mitigate the potential increase in LRI burden in the future (8, 74–79).

This study has several limitations. First, the sudden outbreak of COVID-19 had a profound impact on LRI epidemiological data after 2019. Factors such as whether traditional respiratory pathogens will rebound as non-pharmacological interventions are phased out (8, 74–76), whether altered immune function and vaccination following SARS-CoV-2 infection will affect disease resistance in susceptible populations (77, 78), and whether global attention to these diseases will increase at both individual and national levels following the pandemic all influence future trends in LRIs (80, 81). These uncertainties underscore the need for larger-scale and longer-term studies to capture the full impact of COVID-19 on LRI trends. Second, while the Global Burden of Disease (GBD) database provides valuable data on the overall burden of disease, it has inherent limitations. The GBD data are derived from multiple sources, such as epidemiologic studies, hospital reports, and national health surveys, which may vary in quality and coverage (1). As a result, data accuracy may differ across regions, especially in areas with limited resources or inadequate data collection systems, which can affect the reliability of the results. Additionally, GBD data are often supplemented and adjusted through modeling techniques, which can introduce bias depending on the assumptions used and the accuracy of input data. The timeliness of the data is another consideration, as data from earlier years may not fully capture current disease prevalence. Furthermore, the GBD database offers national-level data, but regional data for China were not available, limiting our ability to analyze differences between areas such as Wuhan, Hubei Province, and other regions. Finally, due to the seasonal variations in LRI incidence, monthly data might provide a more accurate reflection of these fluctuations. Given these data limitations, caution is needed when interpreting the results, and we recommend that future studies incorporate localized primary data or high-quality prospective data to complement the GBD data, offering a more comprehensive understanding of the burden of lower respiratory tract infections and a more accurate basis for public health interventions.

## Conclusion

In conclusion, we identified trends in LRIs in China and globally from 1990 to 2021 and assessed the impact of the COVID-19 pandemic on disease incidence. Compared with the world average, China has achieved better results in reducing the burden of disease in LRIs, but the gap between the two will gradually narrow or even reverse in the next 15 years, which requires our attention. Young children and the old adults remain the main burdens of disease, but the old adults are more affected by disease than are children. Strategies to further reduce the burden of LRIs should focus on enhancing vaccination coverage, improving air quality, and strengthening health education to address behavioral risk factors such as smoking. Furthermore, the COVID-19 pandemic has highlighted the importance of robust healthcare systems and preparedness for respiratory disease outbreaks, which should remain a priority in future public health policies.

## Data Availability

The original contributions presented in the study are included in the article/Supplementary material, further inquiries can be directed to the corresponding authors.

## Supplementary material

Supplementary Figure 1 Age-specific incidence **(A)**, mortality **(C)**, DALY **(E)**, and age-standardized rates **(B, D, F)** of LRIs in China in 1990.

Supplementary Figure 2 Age-specific incidence **(A)**, mortality **(C)**, DALY **(E)**, and age-standardized rates **(B, D, F)** of LRIs globally in 1990.

Supplementary Table 1 All-age cases and age-standardized prevalence, morbidity, mortality, YLL, YLD, and DALY rates of lower respiratory tract infections in China and Global, 2019.

Supplementary Table 2 Joinpoint regression analysis: trends in age-standardized incidence and mortality (per 100,000 population) for both sexes in China and globally, 1990–2021.

## References

[1] GBD 2021 Diseases and Injuries Collaborators. Global incidence, prevalence, years lived with disability (YLDs), disability-adjusted life-years (DALYs), and healthy life expectancy (HALE) for 371 diseases and injuries in 204 countries and territories and 811 subnational locations, 1990-2021: a systematic analysis for the Global Burden of Disease Study 2021. Lancet. (2024) 403:2133–61. 10.1016/S0140-6736(24)00757-8 38642570 PMC11122111

[2] GBD 2021 Lower Respiratory Infections and Antimicrobial Resistance Collaborators. Global, regional, and national incidence and mortality burden of non-COVID-19 lower respiratory infections and aetiologies, 1990-2021: a systematic analysis from the Global Burden of Disease Study 2021. Lancet Infect Dis. (2024) 24:974–1002. 10.1016/S1473-3099(24)00176-2 38636536 PMC11339187

[3] GBD 2019 LRI Collaborators. Age-sex differences in the global burden of lower respiratory infections and risk factors, 1990–2019: results from the Global Burden of Disease Study 2019. Lancet Infect Dis. (2022) 22:1626–47. 10.1016/S1473-3099(22)00510-2 35964613 PMC9605880

[4] Falagas ME Mourtzoukou EG Vardakas KZ. Sex differences in the incidence and severity of respiratory tract infections. Respir Med. (2007) 101:1845–63. 10.1016/j.rmed.2007.04.011 17544265

[5] Ursin RL Klein SL. Sex differences in respiratory viral pathogenesis and treatments. Annu Rev Virol. (2021) 8:393–414. 10.1146/annurev-virology-091919-092720 34081540

[6] Shoar S Musher DM. Etiology of community-acquired pneumonia in adults: a systematic review. Pneumonia. (2020) 12:11. 10.1186/s41479-020-00074-3 33024653 PMC7533148

[7] GBD 2021 Risk Factors Collaborators. Global burden and strength of evidence for 88 risk factors in 204 countries and 811 subnational locations, 1990–2021: a systematic analysis for the Global Burden of Disease Study 2021. Lancet. (2024) 403:2162–203. 10.1016/S0140-6736(24)00933-4 38762324 PMC11120204

[8] Chow EJ Uyeki TM Chu HY. The effects of the COVID-19 pandemic on community respiratory virus activity. Nat Rev Microbiol. (2023) 21:195–210. 10.1038/s41579-022-00807-9 36253478 PMC9574826

[9] To KK-W Sridhar S Chiu KH-Y Hung DL-L Li X Hung IF-N. Lessons learned 1 year after SARS-CoV-2 emergence leading to COVID-19 pandemic. Emerg Microbes Infect. (2021) 10:507–35. 10.1080/22221751.2021.1898291 33666147 PMC8006950

[10] Lai CC Wang CY Hsueh PR. Co-infections among patients with COVID-19: The need for combination therapy with non-anti-SARS-CoV-2 agents? J Microbiol Immunol Infect. (2020) 53:505–12. 10.1016/j.jmii.2020.05.013 32482366 PMC7245213

[11] Islam N Sharp SJ Chowell G Shabnam S Kawachi I Lacey B. Physical distancing interventions and incidence of coronavirus disease 2019: natural experiment in 149 countries. Br Med J. (2020) 370:m2743. 10.1136/bmj.m2743 32669358 PMC7360923

[12] Davis HE McCorkell L Vogel JM Topol EJ. Long COVID: major findings, mechanisms and recommendations. Nat Rev Microbiol. (2023) 21:133–46. 10.1038/s41579-022-00846-2 36639608 PMC9839201

[13] Munro APS Feng S Janani L Cornelius V Aley PK Babbage G. Safety and immunogenicity of seven COVID-19 vaccines as a third dose (booster) following two doses of ChAdOx1 nCoV-19 or BNT162b2 in the UK (COV-BOOST): a blinded, multicentre, randomised, controlled, phase 2 trial. Lancet. (2021) 398:2258–76. 10.1016/S1473-3099(22)00271-7 34863358 PMC8639161

[14] Chuang YC Lin KP Wang LA Yeh TK Liu PY. The impact of the COVID-19 pandemic on respiratory syncytial virus infection: a narrative review. Infect Drug Resist. (2023) 16:661–75. 10.2147/IDR.S396434 36743336 PMC9897071

[15] GBD 2021 Demographics Collaborators. Global age-sex-specific mortality, life expectancy, and population estimates in 204 countries and territories and 811 subnational locations, 1950–2021, and the impact of the COVID-19 pandemic: a comprehensive demographic analysis for the Global Burden of Disease Study 2021. Lancet. (2024) 403:1989–2056. 10.1016/S0140-6736(24)00476-8 38484753 PMC11126395

[16] Kim HJ Fay MP Feuer EJ Midthune DN. Permutation tests for joinpoint regression with applications to cancer rates. Stat Med. (2000) 19:335–51. 10.1002/(SICI)1097-0258(20000215)19:3<335::AID-SIM336>3.3.CO;2-Q 10649300

[17] Yang YLK. Age-Period-Cohort Analysis: New Models, Methods, and Empirical Applications. London: Taylor & Francis (2013).

[18] Luo L. Assessing validity and application scope of the intrinsic estimator approach to the age-period-cohort problem. Demography. (2013) 50:1945–67. 10.1007/s13524-013-0243-z 24072610 PMC5129181

[19] Carstensen B. Age-period-cohort models for the Lexis diagram. Stat Med. (2007) 26:3018–45. 10.1002/sim.2764 17177166

[20] Wagner B Cleland K. Using autoregressive integrated moving average models for time series analysis of observational data. Br Med J. (2023) 383:2739. 10.1136/bmj.p2739 38123181

[21] Bai R Liu Y Zhang L Dong W Bai Z Zhou M. Projections of future life expectancy in China up to 2035: a modelling study. Lancet Public Health. (2023) 8:e915–22. 10.1016/S2468-2667(22)00338-3 37004714 PMC10188127

[22] Jiang Q Zhang C. Recent sex ratio at birth in China. Br Med J Glob Health. (2021) 6:5438. 10.1136/bmjgh-2021-005438 34006519 PMC8137222

[23] Ross I Bick S Ayieko P Dreibelbis R Wolf J Freeman MC. Effectiveness of handwashing with soap for preventing acute respiratory infections in low-income and middle-income countries: a systematic review and meta-analysis. Lancet. (2023) 401:1681–90. 10.1016/S0140-6736(23)00021-1 37121242

[24] Delsors E Monsó F López-Román FJ Menárguez-Puche JF Gonzalez-Barberá M Hukelova H. Changes in antibiotic prescription following an education strategy for acute respiratory infections. NPJ Prim Care Respir Med. (2021) 31:34. 10.1038/s41533-021-00247-7 34083534 PMC8175562

[25] Humer E Jesser A Plener PL Probst T Pieh C. Education level and COVID-19 vaccination willingness in adolescents. Eur Child Adolesc Psychiatry. (2023) 32:537–9. 10.1007/s00787-021-01878-4 34550459 PMC8456192

[26] Barbosa W Zhou K Waddell E Myers T Dorsey ER. Improving access to care: telemedicine across medical domains. Annu Rev Public Health. (2021) 42:463–81. 10.1146/annurev-publhealth-090519-093711 33798406

[27] Hu J Zhou R Ding R Ye DW Su Y. Effect of PM_2.5_ air pollution on the global burden of lower respiratory infections, 1990–2019: a systematic analysis from the Global Burden of Disease Study 2019. J Hazard Mater. (2023) 459:132215. 10.1016/j.jhazmat.2023.132215 37557046

[28] Yang WZ. Dramatic achievements in infectious disease prevention and treatment in China during the past 70 years. Zhonghua Liu Xing Bing Xue Za Zhi. (2019) 40:1493–8. 10.3760/cma.j.issn.0254-6450.2019.12.001 32062906

[29] Honigsbaum M. Revisiting the 1957 and 1968 influenza pandemics. Lancet. (2020) 395:1824–6. 10.1016/S0140-6736(20)31201-0 32464113 PMC7247790

[30] Qin Y Zhao MJ Tan YY Li XQ Zheng JD Peng ZB. History of influenza pandemics in China during the past century. Zhonghua Liu Xing Bing Xue Za Zhi. (2018) 39:1028–31. 10.3760/cma.j.issn.0254-6450.2018.08.003 30180422

[31] Kang L Jing W Liu Q Liu J Liu M. The trends of mortality, aetiologies and risk factors of lower respiratory infections in China from 1990 to 2019: Findings from the Global Burden of Disease Study 2019. J Infect Public Health. (2022) 15:870–6. 10.1016/j.jiph.2022.06.016 35797886

[32] Flerlage T Boyd DF Meliopoulos V Thomas PG Schultz-Cherry S. Influenza virus and SARS-CoV-2: pathogenesis and host responses in the respiratory tract. Nat Rev Microbiol. (2021) 19:425–41. 10.1038/s41579-021-00542-7 33824495 PMC8023351

[33] Sulaiman I Chung M Angel L Tsay JJ Wu BG Yeung ST. Microbial signatures in the lower airways of mechanically ventilated COVID-19 patients associated with poor clinical outcome. Nat Microbiol. (2021) 6:1245–58. 10.1038/s41564-021-00961-5 34465900 PMC8484067

[34] Song C Wu L Xie Y He J Chen X Wang T. Air pollution in China: status and spatiotemporal variations. Environ Pollut. (2017) 227:334–47. 10.1016/j.envpol.2017.04.075 28482313

[35] Lewis D. Air pollution in China is falling—but there is a long way to go. Nature. (2023) 617:230–1. 10.1038/d41586-023-01452-9 37121927

[36] Tang JL Abbasi K. What can the world learn from China's response to covid-19? Br Med J. (2021) 375:n2806. 10.1136/bmj.n2806 34853017 PMC9394586

[37] Lugg ST Scott A Parekh D Naidu B Thickett DR. Cigarette smoke exposure and alveolar macrophages: mechanisms for lung disease. Thorax. (2022) 77:94–101. 10.1136/thoraxjnl-2020-216296 33986144 PMC8685655

[38] Maedel C Kainz K Frischer T Reinweber M Zacharasiewicz A. Increased severity of respiratory syncytial virus airway infection due to passive smoke exposure. Pediatr Pulmonol. (2018) 53:1299–306. 10.1002/ppul.24137 30062859 PMC6175106

[39] Yeligar SM Chen MM Kovacs EJ Sisson JH Burnham EL Brown LA. Alcohol and lung injury and immunity. Alcohol. (2016) 55:51–9. 10.1016/j.alcohol.2016.08.005 27788778 PMC5319482

[40] Ryu S Cowling BJ. Human influenza epidemiology. Cold Spring Harb Perspect Med. (2021) 11:a038356. 10.1101/cshperspect.a038356 32988982 PMC8634793

[41] China NBoSo. The Seventh Population Census of China 2020. (2020). Available at: http://www.stats.gov.cn/tjsj/pcsj/rkpc/7rp/zk/indexch.htm (accessed June 01, 2022).

[42] Press CS. National Bureau of Statistics of the People's Republic of China. China Statistical Yearbook (2018).

[43] Luo M Zhao Z He L Su B Liu W Zhang G. Ethnic disparity in pneumonia-specific mortality among children under 5 years of age in Sichuan Province of Western China from 2010 to 2017. BMC Publ. Health. (2019) 19:1722. 10.1186/s12889-019-8056-7 31870346 PMC6929342

[44] National Health Commission of the People's Republic of China. Action Plan for Healthy Children (2018–2020). (2018). Available at: http://www.gov.cn/gongbao/content/2018/content_5327474.htm (accessed July 9, 2024).

[45] WHO U. Ending Preventable Child Deaths From Pneumonia and Diarrhoea By 2025: the Integrated Global Action Plan for Pneumonia and Diarrhoea (GAPPD). Available at: https://apps.who.int/iris/handle/10665/79200 (accessed May 01, 2023). 10.1136/archdischild-2013-305429 25613963

[46] Cillóniz C Polverino E Ewig S Aliberti S Gabarrús A Menéndez R. Impact of age and comorbidity on cause and outcome in community-acquired pneumonia. Chest. (2013) 144:999–1007. 10.1378/chest.13-0062 23670047

[47] Travis K. China ratifies international tobacco treaty. J Natl Cancer Inst. (2005) 97:1404. 10.1093/jnci/dji356 16204690

[48] Middleton J. From 2011, Smoking Will Be Banned Completely in the Medical and Healthcare System. Clear the Air (2009). Available at: http://tobacco.cleartheair.org.hk/?p=946 (accessed July 9, 2024).

[49] Murray MA Chotirmall SH. The impact of immunosenescence on pulmonary disease. Mediat Inflamm. (2015) 2015:692546. 10.1155/2015/692546 26199462 PMC4495178

[50] Liu Y Zhang Y Zhao W Liu X Hu F Dong B. Pharmacotherapy of lower respiratory tract infections in elderly-focused on antibiotics. Front Pharmacol. (2019) 10:1237. 10.3389/fphar.2019.01237 31736751 PMC6836807

[51] Feng Q Lin S Liu H Yang B Han L Han X. Meta-analysis of whole blood transcriptome datasets characterizes the immune response of respiratory syncytial virus infection in children. Front Cell Infect Microbiol. (2022) 12:878430. 10.3389/fcimb.2022.878430 35493728 PMC9043598

[52] Kampmann B Madhi SA Munjal I Simões EAF Pahud BA Llapur C. Bivalent prefusion F vaccine in pregnancy to prevent RSV illness in infants. N Engl J Med. (2023) 388:1451–64. 10.1056/NEJMoa2216480 37018474

[53] Shiri T Datta S Madan J Tsertsvadze A Royle P Keeling MJ. Indirect effects of childhood pneumococcal conjugate vaccination on invasive pneumococcal disease: a systematic review and meta-analysis. Lancet Glob Health. (2017) 5:e51–9. 10.1016/S2214-109X(16)30306-0 27955789

[54] Tsaban G Ben-Shimol S. Indirect (herd) protection, following pneumococcal conjugated vaccines introduction: a systematic review of the literature. Vaccine. (2017) 35:2882–91. 10.1016/j.vaccine.2017.04.032 28449971

[55] Tanner AR Dorey RB Brendish NJ Clark TW. Influenza vaccination: protecting the most vulnerable. Eur Respir Rev. (2021) 30. 10.1183/16000617.0258-2020 33650528 PMC9488965

[56] Bonten MJ Huijts SM Bolkenbaas M Webber C Patterson S Gault S. Polysaccharide conjugate vaccine against pneumococcal pneumonia in adults. N Engl J Med. (2015) 372:1114–25. 10.1056/NEJMoa1408544 25785969

[57] Gessner BD Jiang Q Van Werkhoven CH Sings HL Webber C Scott D. A public health evaluation of 13-valent pneumococcal conjugate vaccine impact on adult disease outcomes from a randomized clinical trial in the Netherlands. Vaccine. (2019) 37:5777–87. 10.1016/j.vaccine.2018.05.097 29861177

[58] Kraicer-Melamed H O'Donnell S Quach C. The effectiveness of pneumococcal polysaccharide vaccine 23 (PPV23) in the general population of 50 years of age and older: a systematic review and meta-analysis. Vaccine. (2016) 34:1540–50. 10.1016/j.vaccine.2016.02.024 26899372

[59] Qazi S Aboubaker S MacLean R Fontaine O Mantel C Goodman T. Ending preventable child deaths from pneumonia and diarrhoea by 2025. Development of the integrated Global Action Plan for the Prevention and Control of Pneumonia and Diarrhoea. Arch Dis Child. (2015) 100(Suppl.1):S23–8. 10.1136/archdischild-2013-305429 25613963

[60] Sonego M Pellegrin MC Becker G Lazzerini M. Risk factors for mortality from acute lower respiratory infections (ALRI) in children under five years of age in low and middle-income countries: a systematic review and meta-analysis of observational studies. PLoS ONE. (2015) 10:e0116380. 10.1371/journal.pone.0116380 25635911 PMC4312071

[61] Wrenn K Blomquist PB Inzoungou-Massanga C Olufon O Guy RL Hatziioanou D. Surge of lower respiratory tract group A streptococcal infections in England in winter 2022: epidemiology and clinical profile. Lancet. (2023) 402(Suppl.1):S93. 10.1016/S0140-6736(23)02095-0 37997140

[62] Hedberg P. Ventilator-associated lower respiratory tract bacterial infections in COVID-19 compared with non-COVID-19 patients. Crit Care Med. (2022) 50:825–36. 10.1097/CCM.0000000000005462 35148524 PMC9005099

[63] Simon S Joean O Welte T Rademacher J. The role of vaccination in COPD: influenza, SARS-CoV-2, pneumococcus, pertussis, RSV and varicella zoster virus. Eur Respir Rev. (2023) 32:34. 10.1183/16000617.0034-2023 37673427 PMC10481333

[64] Kaur G Danovaro-Holliday MC Mwinnyaa G Gacic-Dobo M Francis L Grevendonk J. Routine vaccination coverage—worldwide. Morb Mortal Wkly Rep. (2023) 72:1155–61. 10.15585/mmwr.mm7243a1 37883326 PMC10602616

[65] Boehm AB Hughes B Duong D Chan-Herur V Buchman A Wolfe MK. Wastewater concentrations of human influenza, metapneumovirus, parainfluenza, respiratory syncytial virus, rhinovirus, and seasonal coronavirus nucleic-acids during the COVID-19 pandemic: a surveillance study. Lancet Microbe. (2023) 4:e340–8. 10.1016/S2666-5247(22)00386-X 36965504 PMC10032662

[66] Cheng S Zhao Y Kaminga AC Zhang X Xu H. China's fight against COVID-19: what we have done and what we should do next? Front Public Health. (2022) 10:548056. 10.3389/fpubh.2022.548056 35844877 PMC9282890

[67] Kang L Li C Du H. Predictors of medical care delay or avoidance among Chinese adults during the COVID-19 pandemic. Patient Prefer Adher. (2023) 17:3067–80. 10.2147/PPA.S436794 38027085 PMC10680038

[68] Yum S Hong K Sohn S Kim J Chun BC. Trends in viral respiratory infections during COVID-19 pandemic, South Korea. Emerg Infect Dis. (2021) 27:1685–8. 10.3201/eid2706.210135 34013875 PMC8153859

[69] Mutnal MB Arroliga AC Walker K Mohammad A Brigmon MM Beaver RM. Early trends for SARS-CoV-2 infection in central and north Texas and impact on other circulating respiratory viruses. J Med Virol. (2020) 92:2130–8. 10.1002/jmv.26010 32410236 PMC7273053

[70] Ghrieb Z Salmona M Michonneau D De Saisset C Allaoua S Kiladjian J-J. Impact of the COVID-19 pandemic on antiviral drug development for other community-acquired respiratory viruses' infections. Therapie. (2023) 78:241–5. 10.1016/j.therap.2022.07.010 36030128 PMC9341168

[71] Wendisch D Dietrich O Mari T von Stillfried S Ibarra IL Mittermaier M. SARS-CoV-2 infection triggers profibrotic macrophage responses and lung fibrosis. Cell. (2021) 184:6243–61.e27. 10.1016/j.cell.2021.11.033 34914922 PMC8626230

[72] Torres-Castro R Vasconcello-Castillo L Alsina-Restoy X Solis-Navarro L Burgos F Puppo H. Respiratory function in patients post-infection by COVID-19: a systematic review and meta-analysis. Pulmonology. (2021) 27:328–37. 10.1016/j.pulmoe.2020.10.013 33262076 PMC7687368

[73] Lechner-Scott J Levy M Hawkes C Yeh A Giovannoni G. Long COVID or post COVID-19 syndrome. Mult Scler Relat Disord. (2021) 55:103268. 10.1016/j.msard.2021.103268 34601388 PMC8447548

[74] Lee SS Viboud C Petersen E. Understanding the rebound of influenza in the post COVID-19 pandemic period holds important clues for epidemiology and control. Int J Infect Dis. (2022) 122:1002–4. 10.1016/j.ijid.2022.08.002 35932966 PMC9349026

[75] Li Y Wang X Cong B Deng S Feikin DR Nair H. Understanding the potential drivers for respiratory syncytial virus rebound during the coronavirus disease 2019 pandemic. J Infect Dis. (2022) 225:957–64. 10.1093/infdis/jiab606 35030633 PMC8807230

[76] Ankert J Hagel S Schwarz C Pan K Wang L von Eiff C. *Streptococcus pneumoniae* re-emerges as a cause of community-acquired pneumonia, including frequent co-infection with SARS-CoV-2, in Germany. ERJ Open Res. (2023) 9:22282988. 10.1101/2022.12.15.22282988 PMC10227630 37260459

[77] Qi S Ngwa C Morales Scheihing DA Al Mamun A Ahnstedt HW Finger CE. Sex differences in the immune response to acute COVID-19 respiratory tract infection. Biol Sex Differ. (2021) 12:66. 10.1186/s13293-021-00410-2 34930441 PMC8686792

[78] Mettelman RC Allen EK Thomas PG. Mucosal immune responses to infection and vaccination in the respiratory tract. Immunity. (2022) 55:749–80. 10.1016/j.immuni.2022.04.013 35545027 PMC9087965

[79] Xiong Y Mi B Panayi AC Chen L Liu G. Wuhan: the first post-COVID-19 success story. Br J Surg. (2020) 107:e431. 32735719 10.1002/bjs.11875 PMC7929289

[80] Kaul V Gallo de Moraes A Khateeb D Greenstein Y Winter G Chae J. Medical education during the COVID-19 pandemic. Chest. (2021) 159:1949–60. 10.1016/j.chest.2020.12.026 33385380 PMC7772576

[81] Babu GR Khetrapal S John DA Deepa R Narayan KMV. Pandemic preparedness and response to COVID-19 in South Asian countries. Int J Infect Dis. (2021) 104:169–74. 10.1016/j.ijid.2020.12.048 33370566 PMC7836380

