# Supplementary figures and images for: Long-term trends and comparison of the burden of lower respiratory tract infections in China and globally from 1990 to 2021: an analysis based on the Global Burden of Disease study 2021
Source: Front Public Health. 2024 Dec 10;12:1507672. doi: 10.3389/fpubh.2024.1507672 (PMC11666531; doi:10.3389/fpubh.2024.1507672)

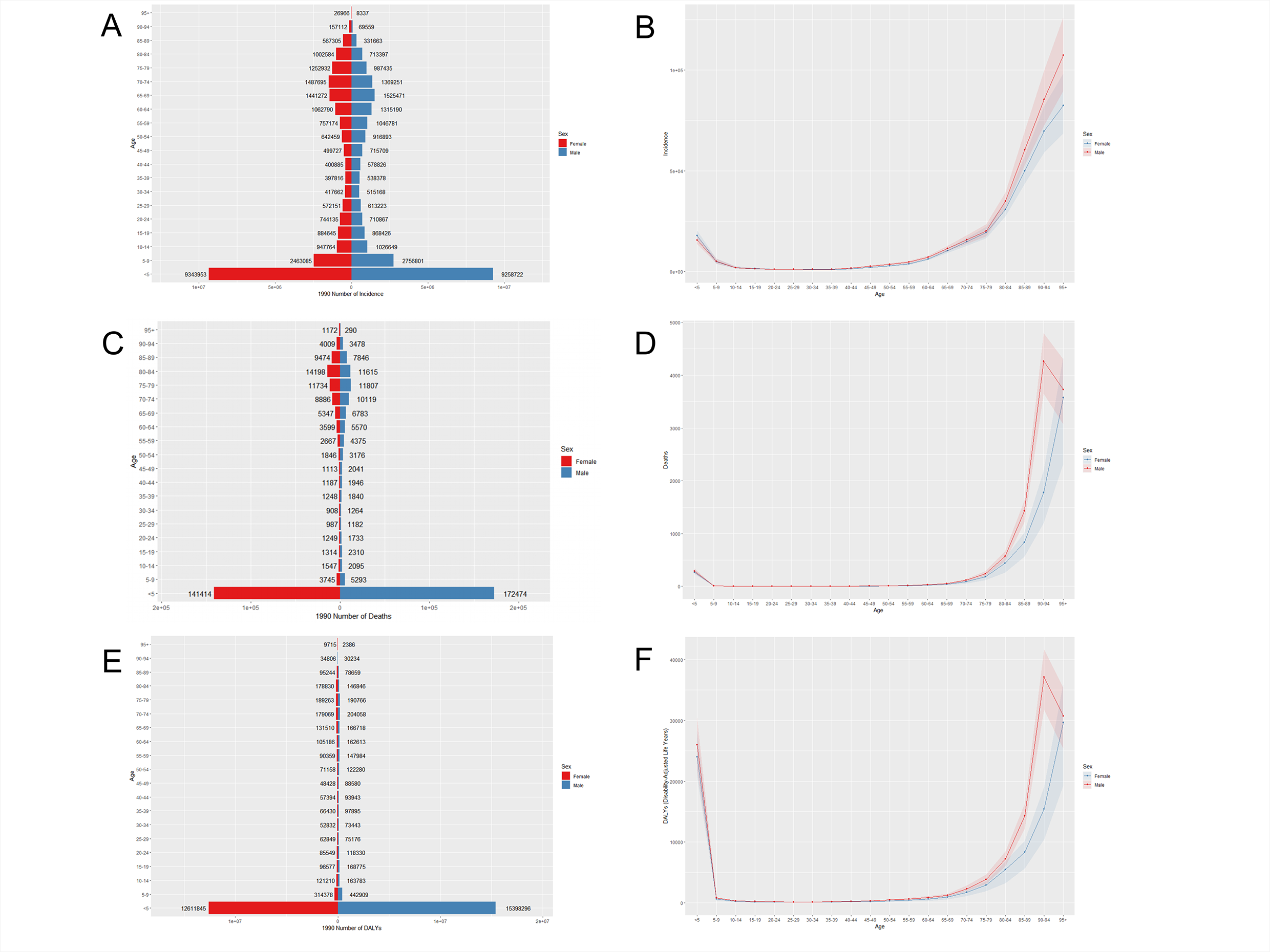

Supplement: Supplementary Figure 1 — Age-specific incidence (A), mortality (C), DALY (E), and age-standardized rates (B, D, F) of LRIs in China in 1990. [file Image_1.tif]

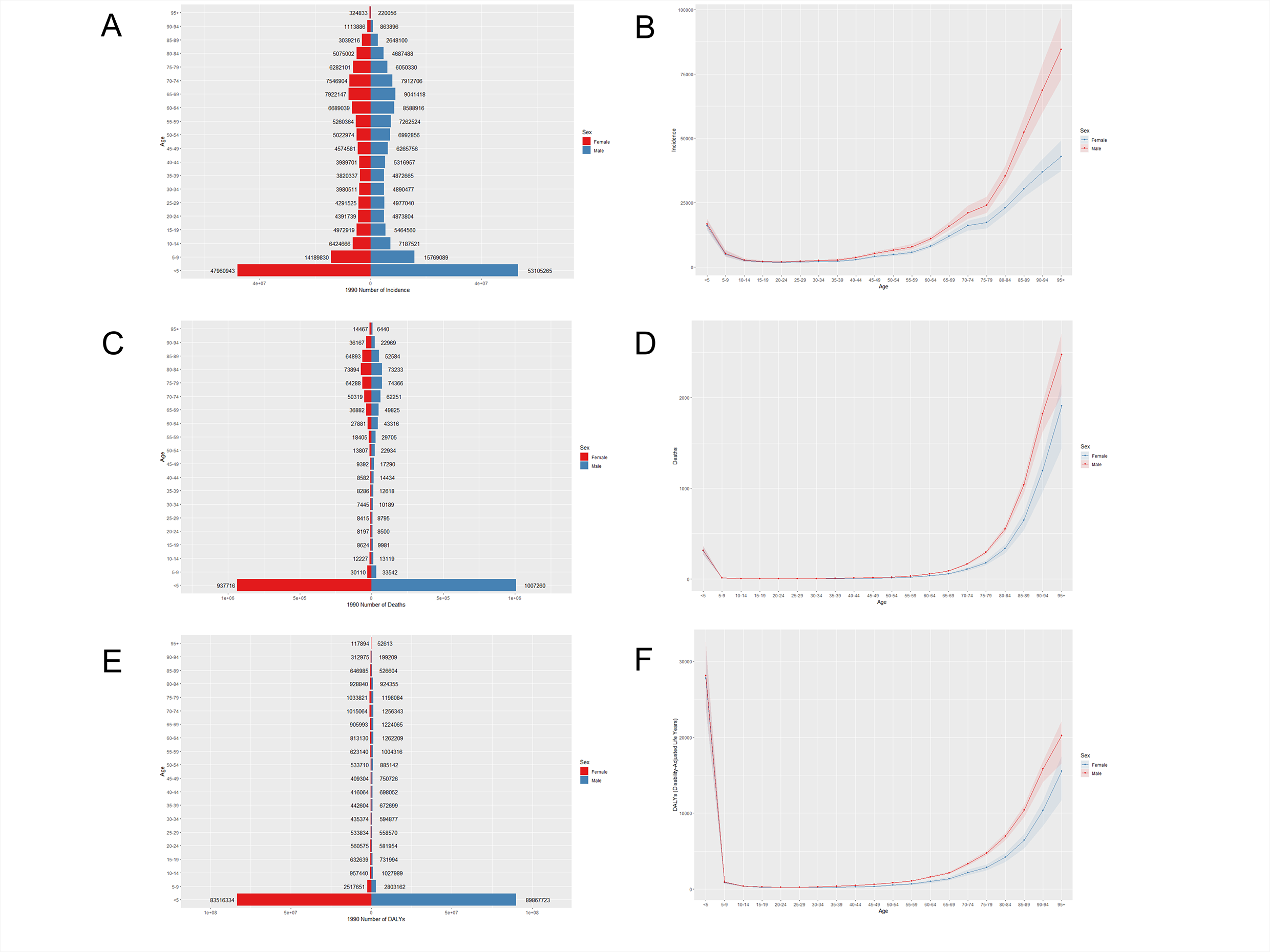

Supplement: Supplementary Figure 2 — Age-specific incidence (A), mortality (C), DALY (E), and age-standardized rates (B, D, F) of LRIs globally in 1990. [file Image_2.tif]
